# Oligomeric Aβ_1-42_ Induces an AMD-Like Phenotype and Accumulates in Lysosomes to Impair RPE Function

**DOI:** 10.3390/cells10020413

**Published:** 2021-02-17

**Authors:** Savannah A. Lynn, David A. Johnston, Jenny A. Scott, Rosie Munday, Roshni S. Desai, Eloise Keeling, Ruaridh Weaterton, Alexander Simpson, Dillon Davis, Thomas Freeman, David S. Chatelet, Anton Page, Angela J. Cree, Helena Lee, Tracey A. Newman, Andrew J. Lotery, J. Arjuna Ratnayaka

**Affiliations:** 1 Clinical and Experimental Sciences, Faculty of Medicine, University of Southampton, MP 806, Tremona Road, Southampton SO16 6YD, UK; S.A.Lynn@soton.ac.uk (S.A.L.); J.A.Scott@soton.ac.uk (J.A.S.); rosie.munday@seh.ox.ac.uk (R.M.); rsd1e14@soton.ac.uk (R.S.D.); E.E.Keeling@soton.ac.uk (E.K.); rw2g14@soton.ac.uk (R.W.); Alex.Simpson@soton.ac.uk (A.S.); davis.dillon1996@gmail.com (D.D.); tf7g11@soton.ac.uk (T.F.); A.J.Cree@soton.ac.uk (A.J.C.); Helena.Lee@soton.ac.uk (H.L.); T.A.Newman@soton.ac.uk (T.A.N.); A.J.Lotery@soton.ac.uk (A.J.L.); 2 Biomedical Imaging Unit, University of Southampton, MP12, Tremona Road, Southampton SO16 6YD, UK; D.A.Johnston@soton.ac.uk (D.A.J.); D.S.Chatelet@soton.ac.uk (D.S.C.); A.Page@soton.ac.uk (A.P.); 3 Eye Unit, University Hospital Southampton NHS Foundation Trust, Southampton SO16 6YD, UK

**Keywords:** retinal pigment epithelium (RPE), amyloid beta (Aβ), age-related macular degeneration (AMD), aging, autophagy–lysosomal pathway, sight loss

## Abstract

Alzheimer’s disease-associated amyloid beta (Aβ) proteins accumulate in the outer retina with increasing age and in eyes of age-related macular degeneration (AMD) patients. To study Aβ-induced retinopathy, wild-type mice were injected with nanomolar human oligomeric Aβ_1-42_, which recapitulate the Aβ burden reported in human donor eyes. In vitro studies investigated the cellular effects of Aβ in endothelial and retinal pigment epithelial (RPE) cells. Results show subretinal Aβ-induced focal AMD-like pathology within 2 weeks. Aβ exposure caused endothelial cell migration, and morphological and barrier alterations to the RPE. Aβ co-localized to late-endocytic compartments of RPE cells, which persisted despite attempts to clear it through upregulation of lysosomal cathepsin B, revealing a novel mechanism of lysosomal impairment in retinal degeneration. The rapid upregulation of cathepsin B was out of step with the prolonged accumulation of Aβ within lysosomes, and contrasted with enzymatic responses to internalized photoreceptor outer segments (POS). Furthermore, RPE cells exposed to Aβ were identified as deficient in cargo-carrying lysosomes at time points that are critical to POS degradation. These findings imply that Aβ accumulation within late-endocytic compartments, as well as lysosomal deficiency, impairs RPE function over time, contributing to visual defects seen in aging and AMD eyes.

## 1. Introduction

The aggregation of intracellular proteins/lipids in the senescent retina is associated with the common irreversible blinding condition, age-related macular degeneration (AMD). These aggregates arise from the incomplete degradation of photoreceptor outer segments (POS) and the accumulation of intracellular lipofuscin within retinal pigment epithelial (RPE) cells [1,2]. The buildup of these macromolecules and related compounds are a major cause of RPE atrophy, which can be assessed by fundus autofluorescence, a biomarker and an accepted clinical end point for the geographic atrophy (GA) form of AMD [3,4]. Extracellular protein/lipid deposits, termed drusen, accumulate under the RPE and are characteristic of early AMD [5]. Histopathological studies of aged donor and AMD eyes also report the accumulation of amyloid beta (Aβ) proteins [6,7,8]. However, their role in retinopathy is less well understood. Aβ accumulates on POS, between the RPE and its supportive Bruch’s membrane (BrM) and in vessels of the underlying choroid. Ultrastructural analysis of drusen has revealed abundant Aβ-containing structures termed ‘amyloid vesicles’ that co-localize with activated complement proteins which also contribute to AMD [9,10]. Aβ co-localized with hydroxyapatite spherules has been reported in sub-RPE deposits [11]. Conformer-specific antibodies have identified different amyloid structures within drusen [12], the most prominent of which consist of Aβ oligomers that were absent in healthy eyes without drusen [13]. The accumulation of sub-RPE Aβ appears common in older individuals and prevalent in those with moderate to high drusen loads [10]. Aβ-positive vesicles within drusen in AMD eyes are most prevalent near GA margins [14]. This distribution of retinal Aβ is recapitulated in wild-type mice [15] and transgenic mouse models of AMD [16,17,18]. Of note, immunization with Aβ antibodies prevented the deposition of sub-RPE Aβ and rescued sight loss in mouse models of retinopathy [18,19]. Recent findings also show a 7-fold increase in retinal Aβ in Alzheimer’s disease patients compared to cognitively normal controls [20]. Taken together, these findings implicate retinal Aβ with disease pathways including cellular stress, drusen and chronic local inflammation [9,10,21]. Given this histopathological evidence, we sought to elucidate the dynamic effects of Aβ in living eyes and further investigate how Aβ causes pathology at the cellular level.

To address these questions, we exploited an in vivo model in which human oligomeric Aβ_1-42_ was subretinally injected into wild-type mouse eyes to recapitulate the high Aβ burden in AMD retinas. The development of AMD-like pathology was longitudinally evaluated in living eyes using non-invasive techniques including color fundus photography (CFP), full-field electroretinograms (ERG) and optical coherence tomography (OCT). These studies were complemented with end-point histological analysis of mouse eyes. Pathology in affected tissue layers was investigated further using in vitro models of choroidal endothelial and retinal pigment epithelial cells. The intracellular fate of Aβ was studied in detail as it was rapidly internalized by RPE cells. Our findings reveal that impaired proteolytic mechanisms in RPE cells coupled to the rapid accumulation of Aβ in lysosomes could be a novel pathway of cellular dysfunction. Next, we investigated potential effects of Aβ-mediated pathology related to the cells’ ability to traffic photoreceptor outer segments, which are processed daily by the RPE. Our findings not only provide evidence of how Aβ targets specific tissues in the outer retina, but also reveal altogether novel and unexpected mechanisms through which pathology can be initiated at the cellular level, contributing to sight loss in later life.

## 2. Materials and Methods

### 2.1. Preparation of Oligomeric Aβ_1-42_

Oligomeric Aβ_1-42_ was prepared according to previously published methods [22,23]. Briefly, lyophilized human recombinant Aβ_1-42_ (rPeptide, Watkinsville, GA, USA) was reconstituted to 1 mg/mL in HFIP, vortexed for 60 s and sonicated at 50 Hz for 5 min. The resuspension solvent was subsequently removed with O_2_-free dry nitrogen and vacuum desiccation for 30 min. Resultant peptidic films were then suspended in DMSO to a concentration of 1 mg/mL, vortexed and applied along with 40 μL Aβ buffer stacker to a 2 mL, 7K Zeba buffer exchange column pre-equilibrated with Aβ buffer (10 mM C_8_H_18_N_2_O_4_S, 50 mM NaCl, 1.6 mM KCl, 2 mM MgCl_2_·6H_2_O and 3.5 mM CaCl_2_·6H_2_O pH 7.4). Columns were centrifuged at 1000× *g* for 2 min at 4 °C to facilitate buffer exchange and eluates spun at 16,000× *g* for 30 min to remove preformed fibrillar aggregates. Aβ_1-42_ concentrations were determined with Beer–Lambert’s Law using the absorbance measured at A280 with a ND-1000 Nano drop spectrophotometer and the molar extinction co-efficient of Aβ (1490 M^−1^·cm^−1^). Alexa Fluor^®^-tagged Aβ was prepared as described. However, prior to application of Aβ_1-42_ to the exchange columns, 20 μL sodium bicarbonate and 10 μL of 11.3 nm/μL dye dissolved in ddH_2_O were added to Aβ_1-42_ in DMSO and incubated for 15 min at room temperature. Concentrations of Alexa Fluor 488 or 647-tagged Aβ_1-42_ were determined as described above taking into account the respective Alexa Fluor contributions to 280 nm absorbance as stated in the manufacturer’s protocol. Preparations within the range of 50–130 μM were incubated on ice for 1.5 h prior to use in experiments to ensure the highest concentration of oligomeric Aβ_1-42_. Bacterial endotoxin levels in preparations were measured using a Limulus Amebocyte Lysate (LAL) Endosafe PTS cartridge (Charles River Laboratories Inc., Boston, MA, USA), which demonstrated that <0.5 EU/mL was present in samples. This is in line with the limits specified by the US Food and Drug Administration (FDA).

### 2.2. Animal Housing and Husbandry

Animal studies were overseen by the institutions’ Ethical Research Committee and were carried out in accordance with the UK Animal (Scientific Procedures) Act of 1986. Experiments also conformed to the ARVO statement for the Use of Animals in Ophthalmic and Vision Research. Mice were obtained from the Biomedical Research Facility (University of Southampton, Southampton, UK). For non-invasive retinal scans, 13 female test-naïve C57BL/6J mice were used aged 117 ± 4 days and weighing 22.8 ± 0.23 g. Animals were housed in a 12/12 h light–dark cycle at 19–24 °C and allowed access to standard laboratory chow and water *ad libitum*. Conventional cages contained Lignocel 2/2 (IPS Ltd., London, UK) bedding and environmental enrichment, housing no more than 10 mice per cage. All experiments were performed between 9:00 and 18:00 h in the light stage of the light–dark cycle. Animal body weight was monitored throughout experiments as a surrogate measure of welfare and studies terminated if a ˃10% change occurred over a period of 3 days. The sample size was determined using the resource equation method taking into account attrition rates after successful subretinal injections. Mice were euthanized on day 15 post-transscleral subretinal injection by cervical dislocation and cutting of the carotid artery.

### 2.3. Animal Preparation and Recovery

Mice were anaesthetized with 1 mg ketamine (Bayer PLC, Reading, UK) and 0.005 mg dexmedetomine hydrochloride (Centaur Services, Castle Carry, UK) per 10 g weight via intraperitoneal (IP) injection. Ketamine facilitates pupillary dilation and is thus an optimal anesthetic for ocular imaging and functional assessments. To minimize stress, animals were subsequently housed in an environment with minimal lighting until cessation of motor function. Pupils were dilated by application of 1 drop 2.5% *w*/*v* phenylephrine hydrochloride (Chauvin Pharmaceuticals Ltd., London, UK) for 2 min followed by 1 drop of 1% *w*/*v* Tropicamide (Chauvin Pharmaceuticals Ltd., London, UK) for 2 min. Dilated eyes were kept hydrated throughout anesthesia via repeated application of Viscotears (Alcon, Farnborough, UK). During this period, mice were also maintained on a heat pad and their breathing rate was monitored. Animals were revived via subcutaneous injection with 200 μL 0.5 mg/mL antipamezole hydrochloride (Centaur Services, Castle Carry UK), recovered on a heat pad and returned to the home cage once righting reflexes had returned.

### 2.4. Transscleral Subretinal Injection Surgery

Transscleral subretinal injections were carried out via the choroid and BrM without retinal penetration using a Stativ Opmi CS/S4 surgical microscope (Carl Zeiss Ltd., Cambridge, UK). For a visual presentation of transscleral subretinal injection surgery, we refer the reader to Dumitrescu et al. [24]. This method is preferred over intravitreal injections as it not only delivered treatment in close proximity to the photoreceptors and RPE, the main cell types compromised in AMD, but also circumvented post-operative inflammatory complications such as vitritis and endophthalmitis. Under ketamine anesthesia, the globe is prolapsed/proptosed enabling access to the posterior globe. Needles were sterilized in 70% ethanol and sterile water prior to use and were replaced every two mice to prevent blunting. A 6 mm glass coverslip (VWR, Lutterworth, UK) was placed onto the cornea to prevent light diffraction and tooth forceps were used to stabilize the globe and provide counter traction during unilateral transscleral subretinal injection. A beveled 34-gauge Hamilton syringe (Hamilton Company, Reno, NV, USA) was inserted at an oblique angle through the sclera until the tip was visualized under the retina and was slowly advanced forwards parallel to the retina, and side to side, in order to create space for the subretinal injection/bleb. Either 1.5 μL of 625 nM recombinant human oligomeric Aβ_1-42_ (*n* = 7) or vehicle (*n* = 6) was then slowly injected by controlled depression of the plunger. Needles were left in place for 5–10 s afterwards to prevent potential back flow of any fluid. The success of the subretinal injection was confirmed by observing presence of a retinal bleb through the operating microscope. A co-investigator was on hand at all times to refocus the microscope, assist with stabilizing the mouse and visually verify subretinal injection via the second eyepiece on the operating microscope. Both eyes of each animal was subretinally injected with Aβ or vehicle in this manner. Animals with retinal bleeds or perforation of the retina (indicated by a bolus in the vitreous) were excluded from studies. Treatment groups were assigned randomly where the statistical unit was the individual mouse.

### 2.5. Funduscopy

CFP of mouse retinas were obtained using the Micron III Retinal Imaging System (Phoenix Research Labs, Pleasanton, CA, USA). Mice were placed on the imaging platform with corneas aligned with the lens. Images were acquired using a brightfield imaging modality (450–650 nm) and visualized using Micron III Retinal Imaging Microscope Software (Phoenix Research Labs, Pleasanton, CA, USA). In the case of re-imaging retinas at 8 days post-subretinal injection, the optic nerve and retinal veins were used as a reference points to locate the injection site seen immediately post-injection as a retinal ‘bleb’. Further, the entire retina was assessed with the microscope and the lesion area captured to ascertain the full extent of induced pathology.

### 2.6. Full-Field Electroretinography (ERG)

Mice were dark adapted for 12 h prior to ERG recordings and maintained at 27 °C throughout the duration of the procedure. ERG traces were recorded using the Generation II Image-Guided ERG modality attachment to the Micron III Retinal Imaging System (Phoenix Research Labs, Pleasanton, CA, USA) which was housed inside a 6-panel aluminum copper mesh Faraday cage (Micro Control Instruments Ltd., Framfield, UK) to minimize potential electrical interference. Animals were placed on a heated platform and were connected to three electrodes as follows: (1) a ground electrode (inserted into the tail), (2) a reference electrode that was attached to the head, and (3) an electrode with corneal contact, which was achieved by positioning the cornea onto the gold-plated objective lens. ERGs were recorded by stimulation with white LED light (6.8 cd-s/m^2^) of 1.5 mm diameter for 1 ms. Stimulation was performed in two sweeps with a two minute interval from which an average recording was determined. In all cases, oculus dexter measurements were carried out first. Animals were assessed randomly. ERGs were visualized in the V3 Phoenix LabScribe ERG software suite (Phoenix Research Labs, Pleasanton, CA, USA). A-wave and B-wave amplitudes were calculated as the measurement from baseline to the A-wave trough and the A-wave trough to the B-wave peak, respectively. The implicit time (the time interval between stimulus onset and the wave peak) for both the A and B waves was also recorded.

### 2.7. Optical Coherence Tomography (OCT)

OCT imaging was performed at baseline, day 8 and day 15 following subretinal injection after ERGs using the Envisu R2200 VHR SDOIS Mouse Imaging system (Bioptigen Inc., Durham, NC, USA). Mice were wrapped in a surgical swab, loaded into the imaging platform and the eye of interest positioned in line with the mouse retina lens using the multiaxial rodent alignment stage apparatus. The cornea was cleaned with surgical spears and Systane lubricant eye drops (Alcon, Camberley, UK). Cross-sectional previews as well as real-time en face fundus images were used for final alignment with the OCT probe in the InVivoVue Clinic software (Bioptigen Inc., Durham, NC, USA). Images were centered round the optic nerve head before 1.4 mm volumetric scans were acquired through a 50 degree field of view comprising 100 B scans, each of which consisted of 1000 A scans. The whole retina scan function was first employed to confirm that the captured region encompassed the lesions and site of subretinal injection. This was evident in both en face and cross-sectional previews of Aβ-injected mice, but less so in vehicle-injected mice despite scanning the entire retina (see Supplementary AVI Files). Finally, OCT images were segmented for the Retinal Nerve Fiber Layer (RNFL), GCL, INL, OPL, ONL, IS, OS, photoreceptor end tips (ETPRS) and the RPE using the InVivoVue 2.4 Diver automated analysis software (Leica Microsystems, Milton Keynes, UK). Segmentation was performed up to a distance of 476 μm from the ONH at 24 retinal locations where at least one point of the segmentation grid was positioned on the optic nerve. Measurements were averaged to acquire representative thicknesses for individual retinal layers. The total retinal thickness was also recorded by combining values from layers spanning RNFL to the RPE. The lesion volume was calculated in Aβ-injected mice by measuring the maximal width and height of pathology within B scans (spanning the lesion) to obtain an average area. Volumetric measurements were calculated taking into account the number of B scans spanning the lesion at 0.14 mm intervals.

### 2.8. Histological Analysis

Mice were enucleated at one week post-injection and eyes fixed in 4% PFA for 30 min at 4 °C, washed in 1xPBS and dehydrated through a series of sucrose gradients as follows: 5% (60 min), 10% (30 min), 12.5% (30 min), 15% (30 min) and 20% (overnight). Posterior ocular sections comprising the sclera, RPE/choroid and retina were subsequently embedded in optimal cutting temperature (OCT) medium and sectioned at 16 μm sections for hematoxylin and eosin staining (H&E). Slides were dried for 1 h and incubated with Gill’s III hematoxylin for 10 min, tap water for 8 min, 0.03% acid alcohol for 8 min, tap water for 8 min, 0.5% eosin for 1 min, distilled water (30 s) followed by incubation in 50%, 70%, 90% and 100% EtOH with 30 s at each step. Slides were placed in Xylene for 5 min and mounted with DPX mounting medium. Imaging were acquired using an Olympus dotSlide virtual microscopy system (Olympus, Southend-on-Sea, UK) and visualized using the OlyVIA software suite (Olympus, Southend-on-Sea, UK). Tissues were assessed within a 100 μm × 300 μm region of interest at 200 μm intervals and a binary score of 1 or 0 assigned to inner segments (IS), outer segments (OS) and RPE/Choroid to indicate retinal pathology. A total of 15 sections were analyzed per eye from 5 slides at 240 μm intervals (3 sections/slide). Tissues within a 200 μm radius from the injection site was excluded to prevent any effects of mechanical injury caused by the subretinal injection being included in the analysis. This was identified as a ‘knick’ in the tissue extending through the sclera, choroid and RPE, with evidence of more trauma than surrounding regions. This could also be seen as a discontinuity, or break in pigmented retinal layers along with damage to the outer neural retina.

### 2.9. Cell Culture

ARPE-19 cells in passages 23–26 (ATCC) were cultured in Dulbecco’s modified Eagle medium with 4.5 g/L L-D glucose, L-glutamine and pyruvate (DMEM, Invitrogen, Dartford, UK) supplemented with 1% heat inactivated FCS (Gibco, Dartford, UK) and 100 units/mL penicillin, 0.1 mg/mL streptomycin (Sigma Aldrich, Gillingham, UK) in a 37 °C humidified incubator with an atmosphere of 5% CO_2_. Cells were passaged at a ratio of 1:2 with a complete media change every 2–3 days. Cells cultured on 24 mm, 0.4 μm PET Transwell^®^ Permeable Supports were maintained in 2 mL apical and 3 mL basal media volume with a 100% and 20% media change every 2–3 days, respectively [25,26]. RF/6A cells (ATCC) were grown in Ham’s F12 medium (Gibco, Dartford, UK) supplemented with 5% FCS and 100 units/mL penicillin, 0.1 mg/mL streptomycin (Sigma Aldrich, Gillingham, UK) under similar conditions with a media change performed every 2–3 days. RF/6A cells were passaged 1:3 at 80% confluence (~5 every days).

### 2.10. Endothelial Cell Migration Assay

RF/6A choroidal endothelial cells (CECs) were seeded at 1.9 × 10^5^ in 13 mm glass coverslips and cultured until 80% confluence (~5 days). A scratch was created at the center of coverslip using a sterile pipette. Reference points were etched to ensure that identical fields of view were imaged. Cells were washed with 1xHBSS to remove cellular debris, 1 μM Aβ_1-42_ or an equivalent volume of vehicle was applied and cells returned to the incubator for 0, 6, 24 or 48 h. Images were taken using the EVOS-XL Core imaging system across three biological replicates at x4 magnification. To account for any differences in scratch size between wells, data were normalized to the 0 h time point and converted into a percentage (the gap at 0 h was 0%, whilst full closure of the scratch equated to 100%). Scratch closure was quantified by an investigator blinded to the identity of images using ImageJ (NIH, USA) software. The cell migration rate (μm/h) was calculated using the formula V_migration_ = |slope|/2xI [27].

### 2.11. Enzyme-Linked Immunosorbent Assays (ELISA)

Cells were seeded at 5 × 10^4^/well in 24 mm sized Transwell inserts and maintained for a minimum of 4 months prior to use. Secreted levels of human Vascular Endothelial Growth Factor (VEGF), Pigment Epithelium-Derived Factor (PEDF) and Aβ (Aβ_28_, Aβ_40_, Aβ_42_) were quantified in conditioned media from apical and basal compartments at 48 h after a complete media change (*n* = 3) using the following assays: Novex^®^ human VEGF (KHG0111, Life Technologies, Warrington, UK), human PEDF (RD191114200R, Biovendor, Heidelberg, Germany) and Aβ_1-X_ (27729, IBL, Fujioka-Shi, Japan). Assays were carried out according to the manufacturer’s instructions. Three technical replicates were assessed per biological replicate and protein concentrations determined by measuring the absorbance at 450 nm with a FLUOstar Optima microtiter plate reader (BMG LABTECH, Aylesbury, UK).

### 2.12. FITC-Dextran Diffusion Assay

An amount of 1 μM Aβ_1-42_ was applied to 4 month old ARPE-19 cells cultured on 0.4 μm PET Transwells for either 2, 24 or 48 h. The cultures were washed in fresh medium and 500 μL of 1.7 mg/mL 40 KDa FITC-dextran was applied to the apical Transwell compartment. Then, 0.5, 1, 3 and 5 h after application, 100 μL of apical and basal media was removed and the amount of FITC-dextran quantified by measuring the absorbance at 492 nm using a FLUOstar Optima microtiter plate reader (BMG LABTECH, Aylesbury, UK). Concentrations in apical and basal compartments were determined relative to the manufacturers’ standard curves.

### 2.13. Confocal Immunofluorescence

Cells were washed in 1 × HBSS, fixed in 4% PFA for 30 min at 4 °C and blocked in 5% NGS in 0.1% PBST for 1 h. Primary antibody was then applied at dilutions detailed in Table 1 at 4 °C overnight. The following day, cells were washed 3× in 0.05% PBST and incubated with secondary antibody prepared in 0.05% PBST for 1 h. Cells were then washed, incubated with 1 mg/mL DAPI for 10 min and washed again before being mounted with Mowiol mounting medium containing Citifluor antifadant. Minor amendments to the aforementioned protocol were made for LAMP1 and Rhodopsin, where the blocking step, primary and secondary antibody incubations were performed in 1% BSA in 3% TBS-Tween and washing in 1 × PBS. Cells were imaged using a Leica SP8 confocal laser scanning microscope (Leica Microsystems, Milton Keynes, UK).

### 2.14. Live-Cell Imaging

For live-cell imaging, cells were plated at 1 × 10^4^ onto 50 μg/mL fibronectin-coated Ibidi glass μ-slides. At ~80% confluence (3 days later), 1 μM Alexa Fluor-tagged Aβ_1-42_ or an equivalent volume of vehicle was applied for 3 h. For studies with LysoSensor Yellow/Blue DND 160, the slides were returned to the incubators for 24 h prior to application of the probe. For studies with Magic Red, the slides were returned to the incubators for 0.5, 3, 24 and 48 h prior to application of the probe. To evaluate lysosomal Aβ_1-42_ localization, 5 μM LysoSensor Yellow/Blue was applied for 5 min, the cells washed and imaged sequentially at ×63 magnification with a Leica SP8 confocal laser scanning microscope. Z-stacks were acquired across three random fields of view from three biological replicates. For analyzing the size of late-endocytic compartments, RPE cultures were labelled with Alexa Fluor-tagged Aβ_1-42_ and LysoSensor Yellow/Blue DND 160 as described before and the diameter of 25 vesicles was measured per field of view. A total of six images were acquired per condition across three biological replicates. To assess the effects of Aβ_1-42_ on lysosomal cathepsin B activity, Magic Red staining solution (x26) was applied at 0.5, 3, 24 or 48 h after Aβ exposure for 30 min at 37 °C with DMSO as a negative control. To assess the Magic Red response to POS, cultures were fed with isolated porcine POS at 4 μg/cm^2^ as described before [28,29] for 3 h in line with timescales used for Aβ studies. Magic Red fluorescence was quantified at similar time points. The cells were washed and imaged as above. A total of 10 fields of view were acquired per treatment group across four biological replicates. Co-localization between the probes and Alexa Fluor-tagged Aβ_1-42_ was quantified using Volocity software (PerkinElmer, Seer Green, UK) which uses the Costes method [30] to quantify fluorescent signals. Images were acquired at an average of 4 lines and a rate of 600 Hz applied to remove noise. Aβ-Alexa Fluor conjugates were carefully selected and imaging parameters set up to prevent spectral overlap or bleed through.

### 2.15. Quantitative PCR

ARPE-19 cells were seeded at a density of 1 × 10^5^ cells/well in 6-well plates and maintained for two weeks to allow confluent cultures to develop. Total RNA was extracted using a Trizol method at 0.5, 3, 24 and 48 h following a 3 h exposure to oligomeric Aβ_1-42_. Briefly, cells were lysed in 500 μL TriIzol and subject to RNA/protein/DNA phase separation by the addition of 200 μL 1-bromo-3-chloropropane followed by centrifugation (12,000× *g*, 15 min). RNA precipitation was by addition of 2 μL RNase-free glycogen and 250 μL isopropanol to RNA-containing supernatants for 20 min followed by centrifugation at 12,000× *g* for 10 min. Contaminants were removed by suspension in 500 μL 75% ethanol and centrifugation as before, after which RNA pellets were solubilized in RNAse free water. RNA yields were determined using a NanoDrop ND-1000 (Thermo Fisher, Dartford, UK). RNA was transcribed into cDNA using the iScript cDNA synthesis kit (Bio-Rad, Watford, UK) and qPCR was performed on a CFX96 Real-Time PCR detection system (Bio-Rad, Watford, UK) using the SYBR green method. The 20 μL PCR reactions consisted of 0.6 μL 10 μM forward and reverse primers, respectively, 6.8 μL UltraPure™ DNase/RNase-Free distilled water (Invitrogen, Dartford, UK), 10 μL iTaq Universal SYBR Green Supermix (Bio-Rad, Watford, UK) and 2 μL of sample cDNA. Details of primers used are shown in Table 2. The reactions were performed as three technical replicates and over three biological replicates. Quantification was performed by normalization to eukaryotic initiation factor 4A2 (EIF4A2) using the ΔCT and 2^−ΔΔCT^ methods. PCR products were resolved on a 1.2% agarose gel alongside a 100 bp DNA ladder at 120 V, 400 mA for 40 min to confirm primer specificity.

### 2.16. Isolation of Photoreceptor Outer Segments (POS)

POS were isolated according to published procedures [31,32,33]. Mouse retinae were extracted, pooled in KCl buffer (0.3 M KCl, 10 mM HEPES, 0.5 mM CaCl_2_ 1 mM MgCl_2_; pH 7.0) with 48% sucrose *w*/*v*, subject to agitation for 2 min and centrifuged at 5000× *g* for 5 min. The resulting supernatant was filtered through sterile surgical gauze into an Eppendorf tube containing an identical volume of KCl Buffer and solutions centrifuged at 4000× *g* for 7 min to isolate POS pellets. Covalent labelling with FITC was achieved by resuspension and 1 h dark rotation of isolated POS with 500 μL labelling buffer (20 mM phosphate buffer pH 7.2, 5 mM taurine with 10% sucrose *w*/*v*) and 150 μL FITC (2 mg/mL FITC isomer in 0.1 Na_2_CO_3_ buffer; pH 9.5) per retina. FITC-POS pellets were obtained by centrifugation at 3000× *g* for 5 min and suspended in DMEM containing 2.5% sucrose *w*/*v* prior to use. Concentrations were determined by BCA assay (Pierce, Dartford, UK) according to the manufacturer’s instructions.

### 2.17. Determination of POS Phagocytosis

ARPE-19 cells were seeded at 1 × 10^4^ in 50 μg/mL fibronectin-coated Ibidi glass bottom μ-slides 2 weeks prior to use in studies as described before [33]. An amount of 1 μM Alexa Fluor 647-tagged Aβ_1-42_ or an equal volume of vehicle was applied to post-confluent cultures for 3 h, washed and returned to the incubator for 23 h [34]. FITC-POS were applied at 4 μg/cm^2^ for 2 h at 37 °C, 5% CO_2_ to facilitate maximum binding with minimal internalization [35], after which cells were washed, returned to the incubator and fixed in 4% PFA for 30 min at 4 °C. This was performed at 4, 8 and 20 h after POS challenge. Selected time points corresponded to the time taken for POS internalization and lysosomal degradation reported previously in ARPE-19 cells [29,36]. Cultures were stained for LAMP1 after which confocal images were acquired across 6 fields of view per treatment group and across two biological replicates using a Leica SP8 laser scanning confocal microscope. Co-localization between LAMP1 and FITC-POS was quantified using Volocity software (PerkinElmer, Seer Green, UK).

### 2.18. Statistical Methods

Statistical analyses were performed using the GraphPad Prism Software (GraphPad, San Diego, CA, USA). Normally distributed data were analyzed using either the Student’s *t*-test or one-way ANOVA with Tukey’s multiple comparisons. Data where there was no indication of a Gaussian distribution were analyzed using the Mann–Whitney test or Kruskal–Wallis test with Dunn’s multiple comparison. Pairwise comparisons were two tailed. Data are expressed as the means ± with (*n*) representing the number of replicates (indicated in figure legends) with statistical significance denoted as * = *p* ≤ 0.05, ** = *p* ≤ 0.01, *** = *p* ≤ 0.001 and *** = *p* ≤ 0.0001.

## 3. Results

### 3.1. Funduscopy and Electrophysiology Studies in Mouse Eyes Exposed to Aβ Showed Signs of Retinal Pathology without Evidence of Impaired Function

**Figure 1:**
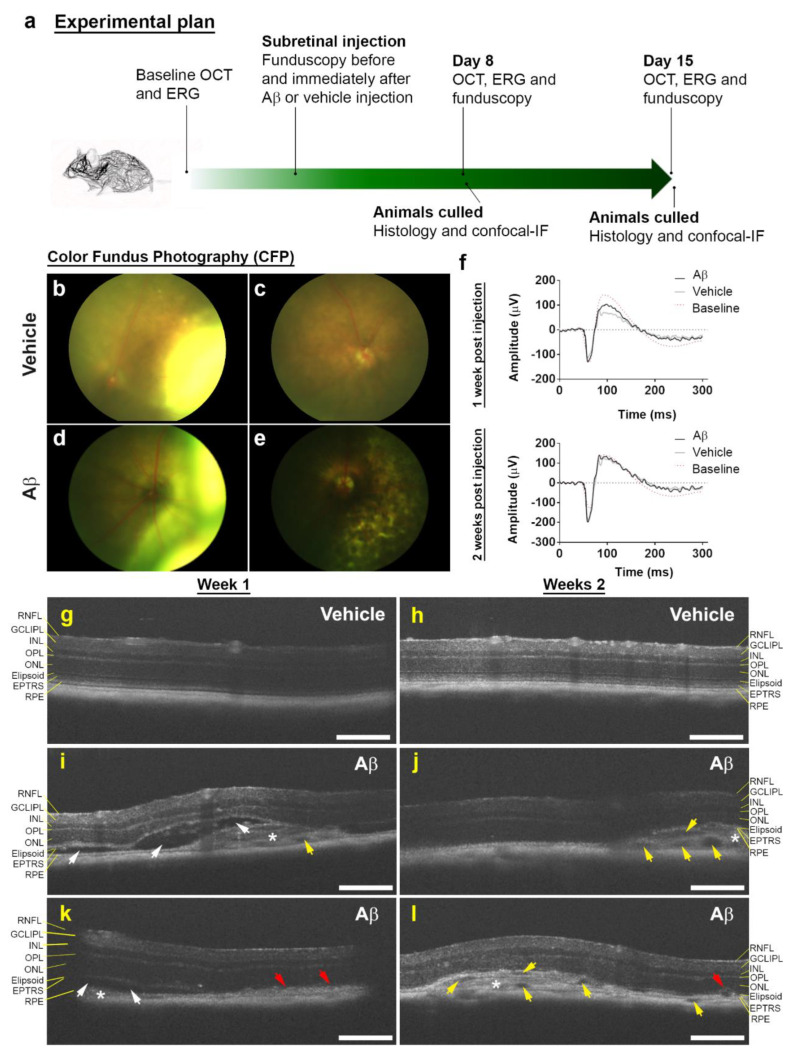
Subretinal Aβ effects in retinas of living mouse eyes. (**a**) Schematic plan showing experimental sequence. (**b**) Representative color fundus photograph (CFP) of mouse eye injected with vehicle immediately after and (**c**) 8 days following subretinal injection. Note, the appearance of a retinal bleb following a successful transscleral subretinal injection, which subsequently resolves. (**d**) Representative CFP of mouse eye injected with human oligomeric Aβ_1-42_ immediately after and (**e**) 8 days later. Note superficial evidence of retinal pathology following exposure to Aβ. (**f**) Average scotopic ERG responses in mice injected with vehicle (*n* = 6) or human oligomeric Aβ_1-42_ (*n* = 7) after 1 and 2 weeks. No significant differences in retinal function were observed between eyes injected with Aβ vs. controls by Mann–Whitney U test (two tailed). (**g**) Representative optical coherence tomography (OCT) images of vehicle and (**i**,**k**) human oligomeric Aβ_1-42_-injected eyes after 1 week. We observed areas of localized pathology in eyes exposed Aβ_1-42_ consisting of RPE disruption (red arrows), subretinal fluid accumulation (white arrows) and hyper-reflective material (asterisk). There was also evidence of occasional hypo-reflective spaces (yellow arrows). However, by week 2, subretinal fluid accumulation appeared to have been largely resolved (**j**,**l**), but there was increasing evidence of hypo-reflective spaces. We also observed disrupted RPE and subretinal hyper-reflective material persisting in week 2. There was no evidence of pathogenic features in eyes injected with vehicle at either 1 or 2 weeks (**g**,**h**). Scale bars correspond to 200 μm.

To study Aβ effects in living eyes, wild-type C57BL/6J mice were subretinally injected with a single dose of human oligomeric Aβ_1-42_. As Aβ levels in ocular fluids are reported to be in the picomolar to nanomolar range [37], we used 1.5 μL of 625 nM Aβ_1-42_ in our experiments. A timeline of the experiment is shown in Figure 1a. Retinae imaged by CFP revealed evidence of pathology in Aβ-injected eyes consisting of patchy pigmentary loss. In contrast, control eyes injected with vehicle appeared normal once retinas had fully reattached following subretinal injection (Figure 1b–e).

We also assessed the functionality of mouse retinas by longitudinal full-field scotopic ERGs. Electrophysiological readouts are routinely used as a measure of overall retinal activity and can provide evidence of functional deficits even in the absence of apparent morphological changes. Alterations associated with retinal aging and disease can therefore be detected by recording the amplitude/duration of A waves (derived from photoreceptor rods/cones) and B waves (derived from the inner retina, predominantly Müller and ON-bipolar cells) after dark adaptation followed by a bright flash. ERG recordings were obtained with measurements set at the widest aperture to encompass the optic nerve and the site of the injection. Results show that within 2 weeks post-exposure, subretinal Aβ accumulation had no effect on global retinal function (Table 3, Figure 1f and Figure S1).

### 3.2. Non-Invasive Scans of Living Mouse Eyes Revealed Evidence of Dynamic Aβ-Induced Pathology in Localized Areas

In parallel, we tested for gross structural changes to mouse eyes using OCT. This non-invasive method provides detailed cross-sectional information on the thickness/structure of individual ocular layers across the scanned area. OCT revealed striking evidence of localized pathology in Aβ-treated eyes including subretinal fluid accumulation, areas of RPE hypertrophy and the presence of hyper-reflective material subretinally 1 week after injection (Figure 1i,k). However, by week 2, the subretinal fluid appeared to have been largely resolved. Nonetheless, signs of RPE hypertrophy and the presence of hyper-reflective material persisted for as long as 2 weeks in Aβ-treated eyes. Interestingly, we observed the appearance of numerous hypo-reflective spaces in the hyper-reflective material (Figure 1j,l). Collectively, these data yielded a picture of dynamic, evolving ocular pathology over a 2- week time course in Aβ-challenged eyes that was localized to discrete areas. By contrast, vehicle-injected eyes showed no evidence of pathology at either 1 or 2 weeks (Figure 1g,h). The thickness of each/individual retinal layer as well as the total thickness of the whole retina was measured and compared between Aβ- vs. vehicle-injected animals (Table 4 and Figure S2).

**Figure 2:**
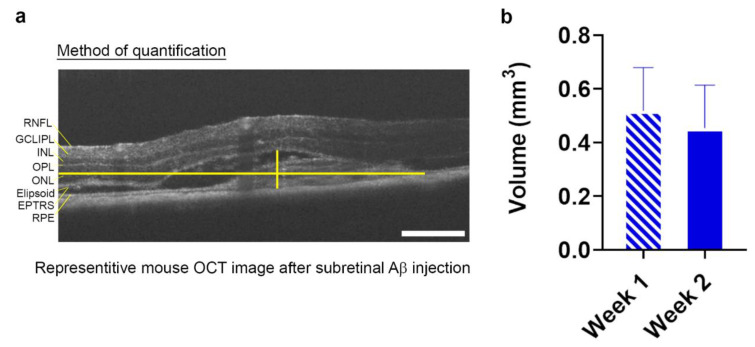
Quantification of Aβ-induced GA-like lesions in living mouse retinae. Optical coherence tomography (OCT) scans of mice subretinally injected with 625 nM human oligomeric Aβ_1-42_ revealed the presence of a discernable focal lesion. (**a**) The maximal height and width of the lesion was measured using the caliper tool function as shown in the sample OCT image and (**b**) presented as volumetric measurements at 1 and 2 week post-injection. The average lesion volume measured 0.52 mm^3^ ± 0.12 SEM at 1 week and 0.45 mm^3^ ± 0.16 SEM at 2 weeks. *n* = 7 mice (*p* = 0.78) Two-tailed Student’s t-test. No significant differences in lesion sizes were observed between weeks 1 and 2.

Our findings show that within a 1.4 mm^2^ scan area, no significant differences were recorded between Aβ- vs. vehicle-injected eyes for any specific retinal layer or indeed for total retinal thickness when measured across the whole retina. Measurement of the lesion volume in each Aβ-injected eye showed a 0.52 ± 0.12 mm^3^ SEM area of damage at 1 week, and an area of 0.45 ± 0.16 mm^3^ SEM at 2 weeks (Figure 2a,b and Supplementary AVI Files of OCT scans).

**Figure 3:**
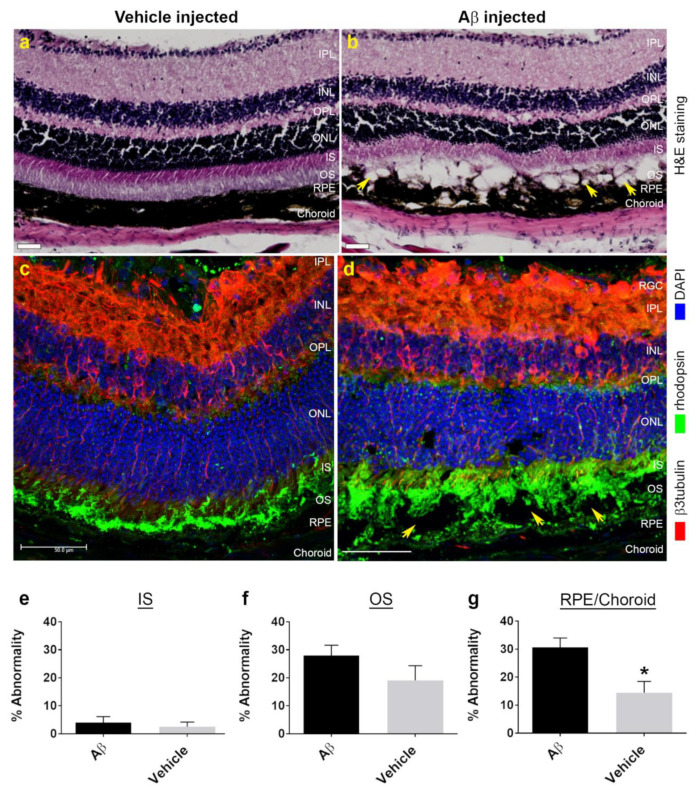
AMD-like histopathology in the outer retina of mouse eyes exposed to human oligomeric Aβ_1-42_ at one week post-injection. (**a**) Hematoxylin and eosin (H&E) staining of tissues from vehicle-injected mouse eyes showed a healthy retina compared to (**b**) eyes exposed to Aβ, where we observed evidence of significant outer retinal disruption. This included diminished inner segments, absence of photoreceptor outer segments as well as a disorganized/atrophic RPE and choroid in a localized area. We also observed the appearance of cystic-like spaces (arrows), which perhaps correspond to areas of subretinal fluid accumulation seen in OCT scans. Scale bars correspond to 50 μm. (**c**) Retinal cross-sections were also probed with anti-rhodopsin to label outer segments (green) and β3-tubulin which labelled retinal neurons (red). (**d**) These studies confirmed the extent of photoreceptor-RPE disruption observed in H&E sections of Aβ-exposed eyes. In contrast, vehicle-injected eyes showed no evidence of any pathology (**c**). Scale bars correspond to 50 μm. Next, we performed line-scan analysis of H&E sections to quantify the extent of Aβ-mediated retinal pathology which is shown as histograms for percentage abnormality on an arbitrary scale. Pathology in Aβ- vs. vehicle-injected eyes was assessed using an unpaired Student’s *t*-test which revealed no differences in (**e**) inner segments, *p* = 0.71 or (**f**) outer segments, *p* = 0.26. (**g**) However, compared to vehicle-treated eyes, we observed a significant level of disruption in the RPE-choroid, *p* = 0.03 in Aβ-exposed eyes. Measurements in *n* = 6 mice for vehicle-injected and *n* = 7 mice for Aβ injected. Error bars represent S.E.M. * denotes a significance of *p* ˂ 0.05.

Animals were culled at predetermined end points and the eyes were processed for histological and confocal immunofluorescence studies. Hematoxylin and eosin staining revealed a pattern of localized retinal pathology consistent with OCT findings. We observed disrupted/atrophic RPE, the absence of photoreceptor outer segments (OS) and disorganized inner segments (IS) (Figure 3b). Discrete cystic spaces were also evident where tissues had parted, likely corresponding to areas of fluid accumulation and hypo-reflective spaces observed during OCT imaging. However, damage was confined to the outer retina, whilst the inner retina appeared to be largely unaffected. There was no evidence of ocular pathology in vehicle-injected animals in weeks following the experiment (Figure 3a). Confocal immunofluorescence studies in which cryo-sectioned Aβ-treated eyes were probed with β3-tubulin and rhodopsin provided further evidence of damage, where widespread disruption was observed at localized areas of the retina–RPE interface (Figure 3d and Figure S3). By contrast, photoreceptor and RPE layers appeared normal in vehicle-treated eyes (Figure 3c and Figure S3). As pathology was confined to the outer retina, we quantified this in blinded histological sections, whereby regions of interest were scored for damage at 200 μm intervals on an arbitrary scale. Findings are reported as average percentage abnormalities for IS: 4.0 ± 2.1 SEM in Aβ-treated eyes and 2.5 ± 1.7 SEM in vehicle controls (Figure 3e). OS: 28.0 ± 3.7 SEM in Aβ-treated eyes and 19.1 ± 5.2 SEM in vehicle controls (Figure 3f). RPE/Choroid: 30.7 ± 3.3 SEM in Aβ-treated eyes and 14.5 ± 4.1 SEM in vehicle controls (Figure 3g). No significant differences were observed between Aβ- vs. vehicle-injected eyes for IS and OS layers, although a pattern indicating diminished OS was observed after Aβ exposure. By contrast, significant disruption was recorded in the RPE/Choroid following Aβ treatment compared to control eyes.

**Figure 4:**
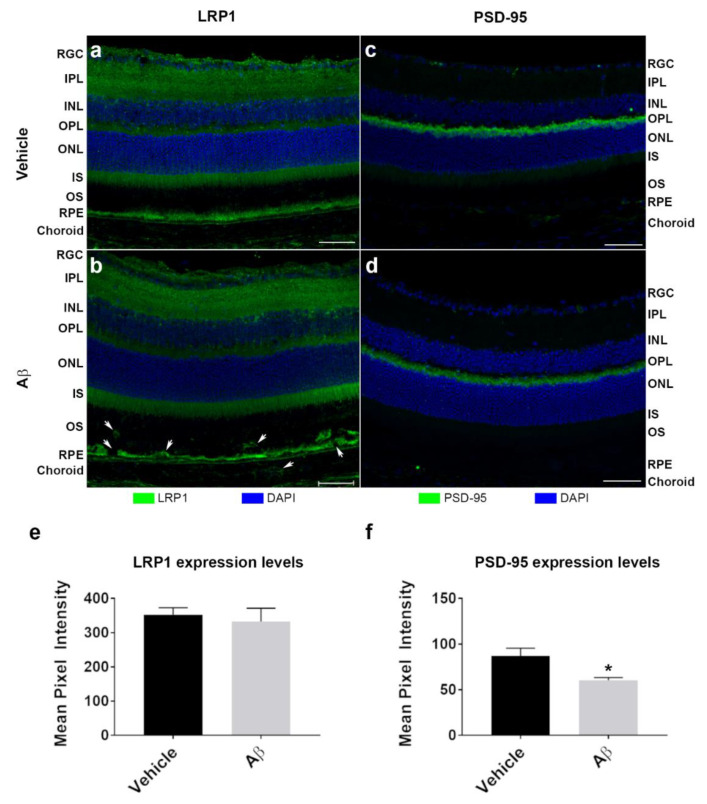
Effects of subretinally injected Aβ on LRP1 and PSD-95 expression in mouse retinas at two weeks post-injection. Mouse eyes injected with human oligomeric Aβ_1-42_ (*n* = 3) or vehicle (*n* = 3) were analyzed to determine potential changes to expression of the Aβ clearance receptor LRP1 and the post-synaptic density marker PSD-95. Two weeks after subretinal injections, animals were culled and ocular cross-sections assessed by confocal immunofluorescence microscopy. (**a**) Representative image shows LRP1 expression in vehicle-injected eye compared to (**b**) eye injected with Aβ. Note, how staining reveals evidence of disrupted OS, RPE/BrM and choroid after Aβ exposure (arrows). (**c**) Representative image shows PSD-95 expression in vehicle-injected eye compared to (**d**) eye injected with Aβ. Structure of the OPL and adjacent layers of the neuroretina appears to be unaffected by subretinal Aβ exposure. Scale bars correspond to 50 mm. (**e**–**f**) The mean pixel intensity was quantified for all layers (retina, RPE/BrM and choroid) and presented as a combined value for each treatment. No changes was observed in LRP1 expression between Aβ and vehicle-injected eyes (unpaired Student’s *t*-test, *p* = 0.67, two tailed). By contrast, expression of PSD-95 was significantly diminished 2 weeks after Aβ treatment compared to control eyes (*p* = 0.04, two tailed). Error bars represent S.E.M. * denotes a significance of *p* ˂ 0.05.

Mouse eyes were also probed for the low-density lipoprotein receptor-related protein 1 (LRP1) to assess whether Aβ had elicited a potential change in the expression of this well-known Aβ-clearance transporter (Figure 4a,b). Diffuse LRP1 expression was observed throughout the neuroretina of Aβ or vehicle-treated eyes with particularly strong expression in the RPE. We also probed for PSD-95 to determine whether Aβ had disrupted synaptic connections between the inner and outer retina (Figure 4c,d and Figure S4). Fluorescence intensities for LRP1 and PSD-95 were recorded in the retina and associated tissue layers and presented as combined average mean pixel values. LRP1: 353.2 ± 19.9 SEM in vehicle control and 333.3 ± 38.7 SEM in Aβ-treated eyes. PSD-95: 87.2 ± 8.4 SEM in vehicle control and 60.4 ± 3.0 SEM in Aβ-treated eyes (Figure 4e,f). No differences in LRP1 expression were observed between control vs. Aβ-treated eyes. By contrast, Aβ exposure resulted in a significant reduction in PSD-95 expression after 2 weeks.

### 3.3. In Vitro Studies Revealed Aβ Effects in Choroidal Endothelial Cells and in the RPE Monolayer

**Figure 5:**
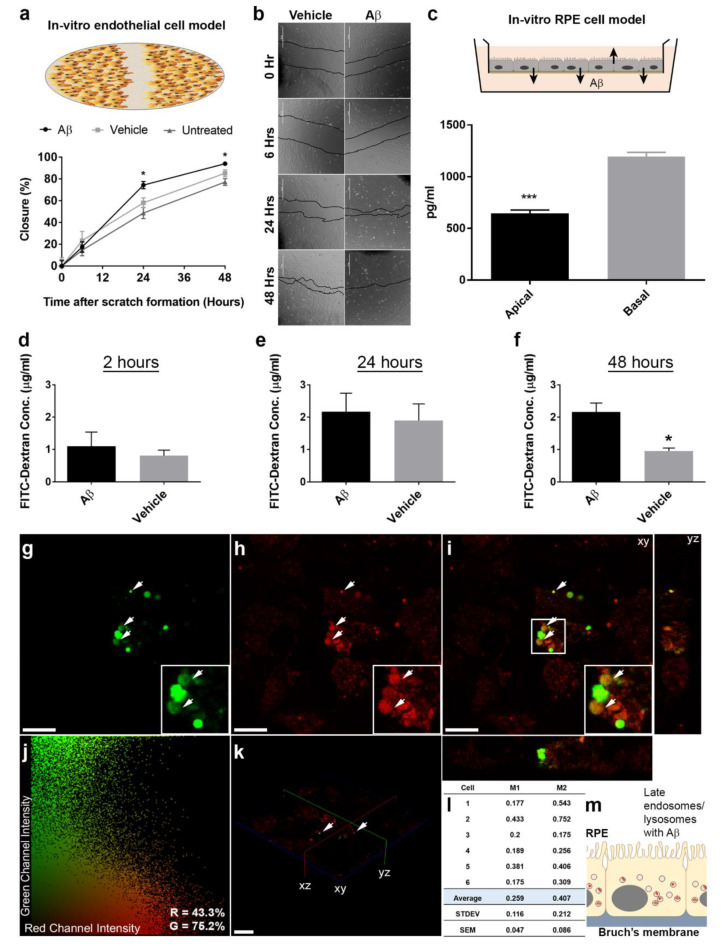
Human oligomeric Aβ_1-42_ directly targets choroidal endothelial and RPE cells. (**a**) The consequences of Aβ exposure on choroidal endothelial cells were studied using a scratch assay. (**b**) Representative bright filed images showing closure of the scratch between 0, 6, 24 and 48 h after treatment with either 1 μM of human oligomeric Aβ_1-42_ or vehicle. Exposure to Aβ resulted in a significantly rapid closure of the scratch at 24 and 48 h compared to vehicle or untreated controls (*n* = 4). One-way ANOVA with Tukey’s multiple comparisons test, where * denotes a significance of *p* ˂ 0.05. (**c**) As the RPE is a major source of retinal Aβ, we next quantified total soluble Aβ_1-x_ levels in an in vitro model. Significantly higher amounts of Aβ was preferentially secreted via the basolateral RPE surface compared to Aβ levels secreted apically (*n* = 3 from 3 independent experiments). Unpaired Student’s *t*-test, where *** denotes a significance of *p* ˂ 0.001. To determine whether exposure to elevated Aβ impaired integrity of the RPE barrier, we quantified the passage of a FITC-dextran substrate from the apical to the basal Transwell chamber at (**d**) 2 h, (**e**) 24 h and (**f**) 48 h. Exposure to 1 μM human oligomeric Aβ_1-42_ resulted in a markedly increased paracellular permeability at 48 h compared to vehicle-treated controls (*n* = 3). Mann–Whitney U test where * denotes a significance of *p* ˂ 0.05. Next, we studied whether exposure to Aβ resulted in its internalization by RPE cells. Representative confocal micrographs showing (**g**) Alex Fluor 488-tagged Aβ_1-42_ (green) with (**h**) LysoSensor DND-160 (red) (**i**) co-localizing in merged en face image (yellow: in white arrows) 24 h after Aβ exposure. Orthogonal views are shown alongside. Scale bars correspond to 40 μm. (**j**) Representative 2D scatter plot (cell 2 in (**l**)) generated by Costes analysis where thresholds are indicated in black along an axis providing a qualitative indication of co-localization. Manders split coefficients are shown in the bottom right. (**k**) 3D projection of cultured RPE cells showing Alex Fluor 488-tagged Aβ_1-42_ in green co-localizing with LysoSensor probe in red. Scale bar corresponds to 40 μm. (**l**) Costes overlap coefficients M1 and M2 indicating percentage of red co-localizing with green, and green co-localizing with red, respectively. Therefore, ~26% (0.259) of lysosomes were positive for Aβ, whilst 40.7% (0.407) of the Aβ signal co-localized to lysosomes. Measurements in *n* = 6 cells across three fields of view. Quantification was performed using Volocity software. (**m**) Schematic showing late-endocytic perinuclear compartments in RPE cells, which were labelled with Aβ_1-42_ in these experiments.

Given the extent of Aβ pathology observed predominantly in the RPE and choroidal layers of living eyes, we used in vitro cultures to understand how Aβ could induce such changes in more detail. First, we tested whether Aβ could directly influence or modify the behavior of choroidal endothelial cells (CECs) using a scratch assay. After exposing CECs to either vehicle or 1 μM of human oligomeric Aβ_1-42_, we recorded the percentage closure of the wound at the following time points: 6 h: 14.5 ± 5.1 SEM untreated, 23.4 ± 8.5 SEM vehicle and 17.5 ± 4.8 SEM Aβ-treated (F_2,32_ = 0.49); 24 h: 48.7 ± 5.2 SEM untreated, 58.1 ± 4.6 SEM vehicle and 74.4 ± 3.3 SEM Aβ-treated (F_2,33_ = 8.52); 48 h: 77.2 ± 3.0 SEM untreated, 85.3 ± 3.1 SEM vehicle and 94.1 ± 0.6 SEM Aβ-treated (F_2,33_ = 11.3). One-way ANOVA with Tukey’s multiple comparisons test. Exposure to Aβ resulted in an increased rate of wound closure with significant differences recorded at 24 and 48 h compared to vehicle or untreated controls (Figure 5a,b).

Next, we used an in vitro RPE model [25,26] to study how Aβ could affect the RPE monolayer. Previous studies had shown that RPE exposed to ≥10 μM Aβ_1-40_ resulted in elevated Vascular Endothelial Growth Factor (VEGF) expression whilst reducing Pigment Epithelium-Derived Factor (PEDF) [38]. We treated RPE monolayers with vehicle or 1 μM of human oligomeric Aβ_1-42_, and quantified the amounts of directionally secreted VEGF and PEDF in apical and basal Transwell compartments after 24 and 48 h (Figure S5). Surprisingly, exposure to physiological amounts of human oligomeric Aβ_1-42_ had no effect on PEDF and VEGF levels secreted by RPE cells compared to the vehicle control. Moreover, PEDF and VEGF secreted by RPE cells were consistent with levels reported in other studies [25,26]. Given that the RPE itself is considered to be a major source of Aβ in the outer retina [6,7,8], we quantified Aβ that was directionally secreted by the RPE. ELISA quantification of total soluble Aβ_1-X_ secreted by cultured RPE over a 48 h period in Transwells yielded values of 644.6 ± 33.3 pg/mL SEM in the apical chamber which were similar to combined Aβ_1-40_ and Aβ_1-42_ levels reported in the human vitreous [39], and 1195.8 ± 39.7 pg/mL SEM in the basal compartment. Our findings revealed that Aβ was preferentially secreted basolaterally towards the choroid at levels approximately 1.8-fold over the amount directed towards the neuroretina (Figure 5c). Given the importance of the blood-retinal barrier to normal retinal function, we sought to determine whether nanomolar concentrations of Aβ could disrupt the RPE monolayer. Confocal immunofluorescence analysis of cultures exposed to 750 nM human oligomeric Aβ_1-42_ identified small/contracted cells, whilst those treated with vehicle retained a characteristic cobblestone RPE morphology. Cell borders were visualized by zonula occludens (ZO-1) labelling, where foci of contracted cells resembling a lesion were particularly noticeable after 48 h following Aβ treatment (Figure S6). In order to assess whether such a compromised barrier led to any functional consequences for the RPE, we assessed the paracellular permeability of cultures following exposure to vehicle or 1 μM of human oligomeric Aβ_1-42_. FITC conjugated dextran was added to the apical Transwell compartment and the fluorescence quantified in the basal Transwell chamber at the following time points: 2 h: 1.1 ± 0.4 μg/mL SEM Aβ and 0.8 ± 0.2 μg/mL SEM vehicle; 24 h: 2.2 ± 0.6 μg/mL SEM Aβ and 1.9 ± 0.5 μg/mL SEM vehicle; 48 h: 2.2 ± 0.3 μg/mL SEM Aβ and 1.0 ± 0.1 μg/mL SEM vehicle; Mann–Whitney U test (Figure 5d–f). A marked difference was observed between groups indicating that Aβ had significantly impaired barrier properties of the RPE monolayer after 48 h.

### 3.4. The Intracellular Fate of Aβ and Cellular Response to Its Accumulation

**Figure 6:**
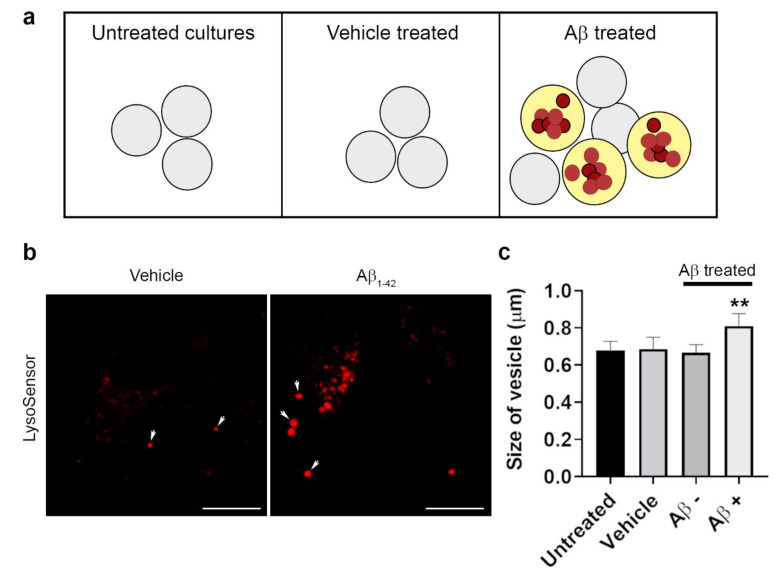
Effect of Aβ on RPE lysosomal size. (**a**) The diameter of LysoSensor-positive vesicles was quantified in RPE cells that were untreated, treated with vehicle or human Aβ_1-42_, 24 h after Aβ exposure. (**b**,**c**) Results show a significant increase in vesicle dimeter in compartments containing Aβ compared to those in the same cell without Aβ cargo or in vehicle-treated or untreated RPE cells. Scale bars in b correspond to 10 μm. The 25 vesicles measured in six random images from three separate experiments per treatment group. A significance of *p* ˂ 0.01 (denoted by **) was observed when the size of Aβ-positive vesicles was compared to vesicle dimeters in all other conditions. One-way ANOVA with Tukey’s multiple comparisons test (F_3,20_ = 8.73).

As Aβ has been shown to accumulate around the RPE in donor aged and AMD tissues [9,10,12,13,14,15], we sought to understand whether it could enter cells. RPE cells specialize in the daily internalization of POS and possess an efficient endo-lysosomal and autophagy pathway in which large quantities of POS are proteolytically degraded [1,40]. We reasoned that if Aβ is internalized by the RPE, they could potentially be trafficked to late-endocytic compartments. RPE cultures were treated with either vehicle or 1 μM of human oligomeric Aβ_1-42_ after which LysoSensor was used to label late compartments in which trafficked cargos are most likely to be found [41]. As cargos are degraded between 16 and 20 h following internalization [29,36], we evaluated the possibility of fluorescently tagged Aβ co-localizing to late-endocytic compartments within this window. Numerous Aβ molecules co-localizing to late endosomes and lysosomes of RPE cells were identified by analysis of confocal immunofluorescence images (Figure 5g–i). The automated, unbiased algorithm [30] used to quantify the extent of co-localization in individual cells showed 25.9 ± 4.7% of the LysoSensor signal (M1: Red channel) co-localizing with Aβ, whilst 40.7 ± 8.6% of the Aβ signal (M2: Green channel) co-localized with the LysoSensor probe (Figure 5j–m). Of note, Aβ was associated with LysoSensor-positive vesicles with an enlarged/swollen phenotype (Figure 5i). We therefore measured the size of these LysoSensor-positive compartments, which revealed a significant increase in the diameter of vesicles with Aβ cargo compared to those in untreated cultures or without Aβ (Figure 6a–c). As these readouts and those from subsequent studies were contingent on a robust link between oligomeric Aβ_1-42_ and its tagged Alexa Fluor probe, we also verified the strength of this conjugation using immunostaining. For this study, RPE cultures were exposed to 1 μM of fluorescently tagged human oligomeric Aβ_1-42_, after which presence of the conjugated molecules were independently verified using the 82E1 antibody. A 48 h time point following Aβ exposure was selected to reflect the longest experimental period within the intracellular environment (Figure S7). Our findings confirmed that all Alexa Fluor tags co-localized with the Aβ 82E1 antibody, indicating that fluorescence was both robust and long-lasting and therefore a genuine indicator of intracellular Aβ molecules in our studies.

**Figure 7:**
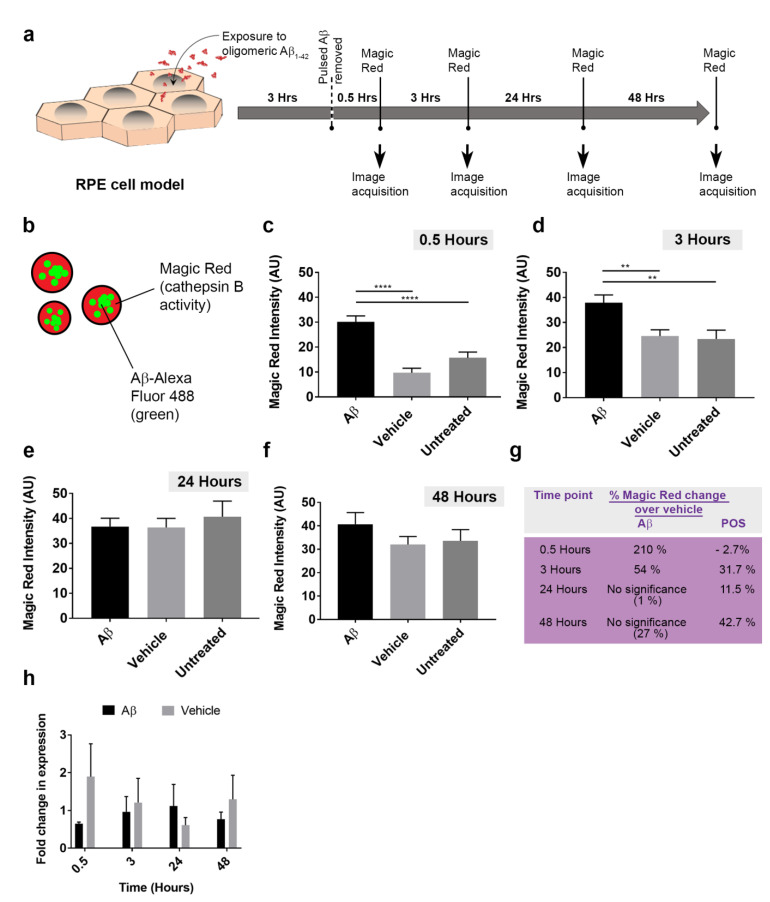
The cellular response to Aβ cargos in RPE lysosomes and transcriptional assessment of cathepsin B activity. (**a**) Schematic showing experimental plan where cultured RPE were exposed to 1 μM oligomeric Aβ_1-42_ for 3 h, following the removal of which Magic Red was used to obtain readouts of lysosomal cathepsin B activity at different time points. (**b**) Schematic showing arrangement of fluorophores in experiment where Aβ-Alexa Fluor 488 (green) and Magic Red (cathepsin B enzymatic activity) can be simultaneously quantified. Time course showing intensity of Magic Red fluorescence at (**c**) 0.5 h, (**d**) 3 h, (**e**) 24 h and (**f**) 48 h following exposure to Aβ, vehicle or untreated controls. Significant differences were observed in Magic Red intensity between Aβ vs. vehicle-treated controls as well as untreated sister cultures at 0.5 and 3 h, which diminished thereafter to baseline levels. *n* = 40 for Aβ_1-42_, vehicle and *n* = 30 for untreated cultures across four biological replicates (10 images analyzed per treatment/experiment). Kruskal–Wallis with Dunn’s multiple comparisons where ** denotes a significance of *p* ˂ 0.01, whilst **** indicate *p* ˂ 0.0001. (**g**) A summary table showing Magic Red activity in response to Aβ cargo as a percentage change over vehicle, compared to responses for POS cargo. (**h**) Quantitative PCR analysis of cathepsin B (CSTB) mRNA expression in relation to the EIF4A2 reference gene as fold change in expression, *n* = 3. No significant differences in cathepsin B mRNA levels were detected between Aβ-treated vs. vehicle or untreated cultures.

Given that ˃40% of internalized Aβ_1-42_ co-localized to RPE lysosomes within 24 h of exposure (Figure 5l), we wanted to assess how these endocytic organelles responded to this new cargo. In live-cell imaging studies, RPE monolayers were treated with either vehicle or 1 μM of human oligomeric Aβ_1-42_ and incubated with Magic Red, a fluorometric probe which provides a quantifiable readout of lysosomal cathepsin B activity (Figure 7a). Magic Red fluorescence was recorded at different time points (Figure 7b–f and Figure S9). The percentage change of fluorescence over the vehicle control revealed that Aβ cargos had resulted in a considerable increase in lysosomal cathepsin B activity at 0.5 h (210%) and at 3 h (54%), after which activity returned to baseline levels (Figure 7g). We also assessed how Magic Red responded to the presence of POS cargos at: 0.5 h: 30.0 ± 2.6 SEM (vehicle) and 29.1 ± 2.7 SEM (POS); 3 h: 42.3 ± 4.3 SEM (vehicle) and 55.7 ± 5.7 SEM (POS). 24 h: 47.4 ± 4.5 SEM (vehicle) and 52.8 ± 2.9 SEM (POS); 48 h: 46.8 ± 3.7 SEM (vehicle) and 66.7 ± 3.9 SEM (POS). Two-tailed unpaired Student’s *t*-test. (Figure S10). In contrast to Aβ cargos, cathepsin B activity in response to POS at similar time points were −2.7%, 31.7%, 11.5% and 42.7% respectively (Figure 7g).

Given that cathepsin B activity was upregulated as a consequence of lysosomal Aβ, we investigated whether this response was mediated at mRNA level. As Aβ could influence the expression of established housekeeping genes, a reference gene was selected from a panel after RT-qPCR analyses. Eukaryotic translation initiation factor 4A2 (EIF4A2), mitochondrial cytochrome C1 (CYC1) [42], glyceraldehyde-3-phosphate dehydrogenase (GAPDH) and β-actin (ACTB) reference genes were assessed. Whole cell lysates from Magic Red experiments were used for RT-qPCR studies. Cycle threshold (C_t_) values for individual candidate reference genes were obtained for each time point and combined in a box and whisker plot to reflect the variability of mRNA expression across all time points/conditions. GAPDH: 32.9 ± 0.4 SEM, ACTB: 23.77 ± 0.2 SEM, CYC1: 27.14 ± 0.1 SEM and EIF4A2: 21.1 ± 0.1 SEM (Figure S11). EIF4A2 mRNA levels showed the least variability amongst the markers and was therefore selected as the reference gene. Cathepsin B mRNA levels were quantified in relation to EIF4A2 expression in Magic Red assays. Through this approach, we could evaluate how cathepsin B mRNA levels changed at time points similar to those used to evaluate cathepsin B enzymatic activity. No significant differences in cathepsin B mRNA levels were detected between Aβ-treated vs. vehicle or untreated cultures (Figure 7h), which suggests that upregulation of lysosomal cathepsin B activity was not driven at the genetic level.

### 3.5. The Dynamics of Aβ Internalization and Consequences to Cargo Trafficking Capability of RPE Cells

**Figure 8:**
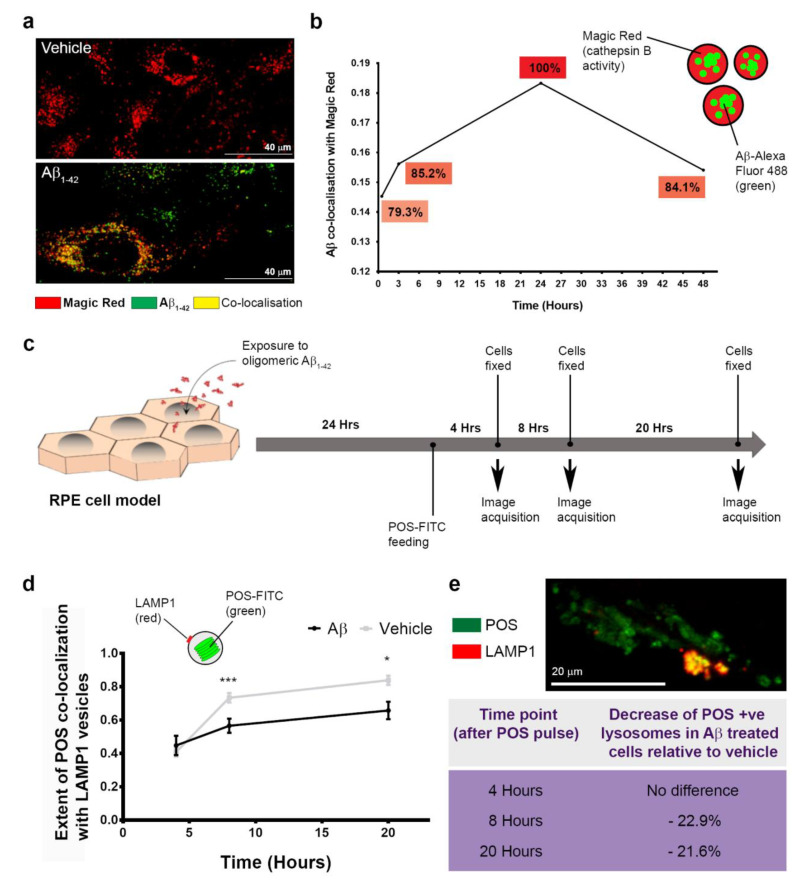
The pattern of Aβ aggregation in RPE lysosomes and consequences to RPE function. (**a**) Representative confocal immunofluorescence image showing a high proportion of late-endocytic compartments positive for Aβ_1-42_ (green), which co-localize with Magic Red to appear yellow. Scale bars correspond to 40 μm. (**b**) Dynamics of Aβ_1-42_ entry into lysosomes of RPE cells shown relative to vehicle-treated cultures, which reached an arbitrary point of maximal aggregation after 24 h. Only minimal degradation of the Aβ fluorescence signal was observed after 24 h following maximal aggregation (or by 48 h after initial Aβ exposure). Consequently, ˃80% of Aβ present at the 24 h time point remained sequestered with RPE lysosomes a day later. Data from three biological replicates. (**c**) Schematic showing experimental plan where RPE cultures exposed to either Aβ_1-42_ or vehicle were fed with POS a day later, and co-localization with LAMP1 vesicles quantified thereafter at 4, 8 and 20 h. (**d**) Graph showing extent of POS co-localization in LAMP1 vesicles after POS feeding. Two-tailed unpaired Student’s *t*-test. Data from three biological replicates, where * denotes a significance of *p* ˂ 0.05, whilst *** indicate *p* ˂ 0.001. (**e**) Representative confocal-immunofluorescence image showing POS-FITC (green) co-localizing with LAMP1 compartments (red) and appear yellow. Scale bar corresponds to 20 μm. Summary table showing percentage decrease in LAMP1-positive vesicles with POS cargos at different time points after POS feeding in Aβ-treated cells relative to vehicle. Aβ-exposed RPE had significantly fewer lysosomes capable to trafficking POS cargos after 8 h.

The Magic Red assay used to evaluate the lysosomal cathepsin B enzymatic response was also used to determine the dynamics of Aβ trafficking into late-endocytic compartments and to quantify [30] Aβ co-localization with Magic Red-positive vesicles (Figure 8a), which is shown relative to vehicle-treated cultures (Figure 8b). At 0.5 h after Aβ treatment, the percentage of fluorescently tagged Aβ co-localized was 79.3%, which increased to 85.2% at 3 h before reaching a maximal value (arbitrary 100%) after 24 h. This declined thereafter to 84.1% by 48 h. These findings revealed that substantial amounts of internalized Aβ_1-42_ persisted within late compartments of RPE cells despite an upregulation of lysosomal cathepsin B.

The daily internalization and proteolytic processing of POS is a key feature of RPE cells [1,40]. We therefore studied whether Aβ could affect the ability of RPE to handle POS cargos. Cultures were treated with vehicle or human oligomeric Aβ_1-42_, 24 h after which they were fed with POS, in keeping with the diurnal cycle (Figure 8c). We then used the algorithm utilized in previous studies [30] to quantify the extent of fluorescent POS molecules co-localizing with the lysosome associated membrane protein 1 (LAMP1) vesicles [41] (Figure 8d). Following POS feeding, quantification was carried out at key time points known to include early, mid and terminal phases of POS digestion [29]: 4 hours: 0.4 ± 0.03 SEM (vehicle) and 0.4 ± 0.06 SEM (Aβ); 8 hours: 0.73 ± 0.03 SEM (vehicle) and 0.6 ± 0.04 SEM (Aβ); 20 hours: 0.8 ± 0.03 SEM (vehicle) and 0.7 ± 0.05 SEM (Aβ). Two-tailed unpaired Student’s *t*-test. Although no differences were noted at the initial 4 h time point, a significant reduction in the number of lysosomes participating in POS handling was observed at 8 and 20 h in cultures exposed to Aβ (Figure 8d,e and Figure S12). These findings revealed a previously unreported mechanism through which Aβ can impair POS trafficking in RPE cells.

## 4. Discussion

This study describes a mouse model where the age-related Aβ burden in the human retina is recapitulated by subretinal injection. Studies of human donor eye tissues as well as mouse models of retinal degeneration show increasing levels of retinal Aβ with advancing age and retinopathy. Many of these studies have reported specific effects of Aβ_1-40_. However, specific changes in Aβ_1-42_ levels in the retina with age and disease is yet to be investigated more fully. One such study assessed Aβ_1-40_ and Aβ_1-42_ concentrations in the human vitreous, but reported no significant association with age [39]. A study of human plasma showed an increasing trend towards higher levels of Aβ isotypes 1-38, 1-40 and 1-42 with advancing AMD. Moreover, an altered Aβ_1-42_ to Aβ_1-40_ ratio showed a robust association with late stages of AMD [43]. Specific effects of Aβ_1-42_ have also been reported in rodent models. Of these, the 5xFAD transgenic model forms one of the most attractive in vivo tools, due to high levels of retinal Aβ [44,45] which can maximize the potential to recapitulate the age-related Aβ pathology observed in human eyes. The 5xFAD mice show intracellular Aβ in RPE cells correlated with decreased ZO-1 and Occludin expression by 8 months [17]. By 10 months, these mice develop characteristic features of GA including RPE vacuoles, hypopigmentation, basal laminar and basal linear deposits as well as BrM thickening and loss of RPE membrane specialization. Microarray analysis of 5xFAD mice also reveal an RPE gene expression profile that is consistent with GA [46]. Collectively, these findings indicate that age- and disease-associated accumulation of Aβ_1-42_ is correlated with worsening retinal pathology. Our findings show localized areas of damage in the RPE and adjacent tissues that share similarities with focal atrophy in GA patients [47]. Use of longitudinal OCT enabled us to capture previously unreported Aβ pathology in living mouse eyes including RPE hypertrophy and the presence of subretinal hyper-reflective material. End-point histological analysis of mouse eyes did not indicate any fibrotic/scar tissue. Instead, evidence of hypertrophic RPE in OCT and histological data suggest an impaired RPE monolayer, likely associated with failure of transport and fluid accumulation. Measurement of the atrophic region revealed the lesion to remain stable for 2 weeks after Aβ exposure. Interestingly, this pattern of localized damage was not evident when individual retinal layers or total retinal thickness was measured across the whole mouse retina. Likewise, potential impairment of retinal function due to localized damage was not detected by full-field ERG, which also occurs in GA patients as focal lesions do not always affect overall retinal function [48]. However, a previous study reported significant differences in A, B and C waves in subretinal Aβ-injected mice [49]. Aβ-mediated pathology in the neuroretina also included diminished synapses in the outer plexiform player (OPL), suggesting potential vulnerability of photoreceptor-bipolar connectivity. Although Aβ does not appear to accumulate in the OPL directly, soluble oligomers are known to impair synapses [50], which may account for the damage to this layer. Previous studies have shown that retinal Aβ is cleared by macrophages [15]. To determine whether alternative mechanisms involved in clearing Aβ could also act in the retina, mouse tissues were stained with LRP1, the major Aβ clearance pathway in the brain [51]. Recent findings from eyes of Alzheimer’s disease patients show a correlation between retinal Aβ accumulation and diminished retinal vascular LRP1 expression [20]. Although we did not focus on retinal vessels *per se*, analysis of the whole retina and associated tissues at two weeks after injection reveal that Aβ was not cleared by this mechanism or alternatively, that this pathway had somehow failed. Additional methods such as immunoblotting and qPCR may be used in future studies to verify these findings. In summary, the pattern of Aβ-mediated retinal damage recapitulated features of focal GA lesions, which only the Southampton AMD model [52,53] and a few other models have successfully re-created [49,54,55,56].

Given the (1) well-established pro-angiogenic properties of Aβ, (2) the presence of choroidal Aβ staining observed in AMD tissues, as well as (3) Aβ effects in the choroid reported in other animal models [57], we sought to understand the mechanisms of Aβ-mediated choroidal pathology using cultured CECs. Our studies revealed that physiological oligomeric Aβ_1-42_ quantities enhanced cell migration, although whether it also triggered cell division is not clear. Next, we investigated effects of the same Aβ species/conformation in RPE cells using ≤1 μM quantities which are comparable to reported ocular Aβ concentrations [37,39]. Exposure of RPE to Aβ_1-42_ has been shown to trigger oxidative stress, barrier deficiencies, senescence, and to induce a pro-angiogenic phenotype amongst other pathogenic features [54,56,58,59,60]. Another study exposed RPE to ≥10 μM Aβ_1-40_, which caused significant VEGF upregulation whilst reducing PEDF [38]. Interestingly, our findings revealed that physiological Aβ_1-42_ quantitates did not affect secreted PEDF or VEGF levels in RPE cells. Moreover, the RPE itself synthesized Aβ, which was consistent with the reported expression of major amyloid precursor protein (APP) isoforms [6,9] as well as age-linked increase in Aβ produced in these cells [61]; supporting the theory that the RPE is a major site of ocular Aβ synthesis [6,8]. Our results also showed that a significant proportion of all soluble Aβ was directed towards the choroid. Taken together, our findings indicate that Aβ plays an important role in the choroid, and that Aβ_1-42_ effects appear to be mediated directly on CECs rather than through upregulation of VEGF by RPE cells. Our studies also demonstrate that exposure to physiological Aβ_1-42_ amounts led to contracted actin foci and elevated paracellular permeability in the RPE, consistent with an impaired blood–retinal barrier reported by others [17,54]. Such abnormal RPE monolayers with altered cytoskeletal arrangements are also reported in donor AMD tissues [62].

Our work and that of others has previously shown that Aβ enters neurons and accumulates in lysosomes [23,63]. APP processing also involves their delivery to lysosomes, the impairment of which results in increased intracellular Aβ accumulation [64]. Given these findings and those reporting Aβ aggregation around the RPE in AMD tissues [9,10,12,13,14,15], we sought to clarify whether Aβ could also enter RPE cells. Our reasoning was further supported by findings showing that acidic lysosomal compartments in neurons formed ideal sites for Aβ aggregation in AD brains [65,66], as well as Aβ-positive RPE cells reported in eyes of 5xFAD transgenic mice expressing high levels of retinal Aβ [17]. Our findings show that following exposure to 1 μM oligomeric Aβ_1-42_, as much as ~26% of the lysosomal signal was positive for Aβ after 24 h. The measured diameters of these late-endocytic compartments correlated with the reported size of lysosomes [67]. We found that Aβ-positive vesicles were enlarged, consistent with swollen POS trafficking lysosomes in oxidatively stressed RPE, which we reported previously [29]. Of note, the increase in ratio between vesicles with and without Aβ (1.2 fold) was identical to the ratio between POS-positive lysosomes in healthy RPE vs. their equivalent cargo-carrying counterparts under oxidative stress. This may have implications for trafficking dynamics, as swollen vesicles are associated with cellular stress and reduced diffusive lysosomal motion [67,68]. Next, we studied the cellular responses to lysosomal Aβ accumulation by quantifying cathepsin B activity; an abundant lysosomal enzyme [69] important to RPE function [70,71]. A time course analysis of Magic Red fluorescence revealed contrasting differences in cathepsin B responses to POS vs. Aβ cargos. There was an immediate and significantly larger response to Aβ at 0.5 h compared to POS. However, 3 h after exposure, the cathepsin B reaction to Aβ had diminished by ~25%, whilst lysosomal enzymatic activity was still increasing in response to POS cargos. At 24 and 48 h, there were no differences in cathepsin B levels in response to Aβ compared to baseline activity. Our findings revealed divergent lysosomal enzymatic reactions to different cargos types, revealing contrasting proteolytic responses by RPE cells. This may be due to the speed of cargo internalization, their biophysical characteristics, or indeed their relative quantities, as the Costes method [30] does not reveal the actual amount of cargo that co-localize with Magic Red fluorescence. However, we speculate the exaggerated response to Aβ may be due to its rapid entry as well as its propensity to aggregate in late acidic compartments to cause lysosomal labialization, reported in other cell types [23,63,65,66,72]. This contrasts with the more measured enzymatic response to POS cargos, which occur on a daily basis in RPE cells [36,40,73]. The modest cathepsin B response to POS should be considered in the context that lysosomes contain ˃60 different hydrolases [67], which we have not measured but could also react to these cargos. The bi-phasic lysosomal cathepsin B responses to two different cargo types in RPE cells were consistent with its enzymatic activity reported in other cells [74]. A similar biphasic response was also observed during collagen IV degradation by cathepsin B, which reportedly regulates proteolytic mechanisms as either an exopeptidase at low levels, or as an endopeptidase at high concentrations [75]. Our findings also revealed that upregulation of lysosomal cathepsin B, which is one of the major cysteine proteases in the RPE [76], was mediated at the post-transcriptional level.

**Figure 9:**
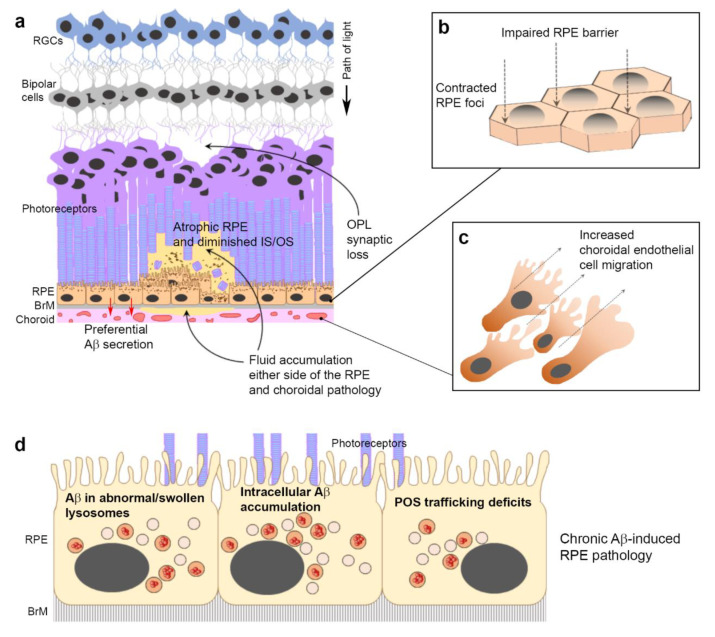
Summary diagram of Aβ-induced pathology in the retina. The age-related Aβ accumulation reported in sub-RPE/drusen, POS and choroid of donor AMD tissues and in animal models is recapitulated by Aβ-injected mice and by in vitro studies. (**a**) Development of localized retinal pathology with GA-like features alongside synaptic loss in the OPL following subretinal Aβ injection in living mouse eyes. (**b**) Impaired RPE barrier with development of contracted RPE foci following exposure to human oligomeric Aβ_1-42_. (**c**) Choroidal pathology in the form of increased endothelial cell migration after Aβ exposure. Aβ effects appear to be mediated directly rather than via upregulation of VEGF. (**d**) Aβ is internalized by RPE lysosomes, which become swollen. An insufficient lysosomal cathepsin B response contributes to Aβ accumulating in RPE lysosomes. RPE exposed to oligomeric Aβ exhibit lysosomal deficits affecting the capacity to traffic/degrade POS cargos, which over time, could contribute to developing visual defects.

Next, we studied whether this lysosomal response successfully cleared Aβ cargos. Our findings showed that Aβ entry into lysosomes occurred over a prolonged period, with a maximal aggregation point at 24 h. However, clearance of Aβ cargos thereafter was slow, with only 15.9% of the maximal cargo cleared in the subsequent 24 h period. This revealed the following salient points. (1) The rapid and substantial burst of lysosomal cathepsin B activity was somewhat out of step with prolonged Aβ aggregation in these compartments. (2) Aβ clearance by cathepsin B, and presumably other lysosomal enzymes, removed only a small proportion of these cargos at least within assay timelines. (3) Prolonged Aβ aggregation in lysosomes and their slower clearance could result in the gradual build-up of Aβ within RPE cells over long periods. Indeed, the uptake of extracellular Aβ at low levels and their concentration as high molecular weight species in endosomes/lysosomes have been reported in neurons affected by Alzheimer’s disease [65]. These findings contrast with the efficient clearance of POS by RPE, which we and others have shown to be largely completed within a 24–48 h period [29,33]. The functional implications of Aβ exposure to RPE activities were evaluated using a POS feeding assay, which revealed a ˃20% reduction in the number of LAMP1-positive lysosomes with POS cargos. Crucially, this deficiency in POS trafficking corresponds to critical timeframes where these cargos are typically degraded in terminal compartments of the proteolytic pathway [29,40]. We attribute the deficiency of POS-trafficking lysosomes to intracellular Aβ, which were internalized rapidly and retained in substantial quantities. This is supported by the observation that both Aβ-treated and control cultures were pulsed with equal POS loads, after which we found no evidence of lysosomal trafficking defects at 4 h. Our findings are consistent with the behavior of intracellular oligomeric Aβ_1-42_, which persisted in lysosomes of neurons for several days to impair their activities [72]. Although each mammalian cell is thought to contain several hundred lysosomes [67], deficiencies in the timely availability of lysosomes for efficient POS degradation could contribute to the accumulation of intracellular macromolecules, a well-defined pathway of RPE atrophy that can be assessed in AMD patients [1,3]. Our results are also in line with findings showing elevated LAMP2 in aged wild-type mice compared to younger animals. Moreover, genetic ablation of LAMP2 resulted in AMD-like pathology including defects in the autophagy–lysosomal pathway [77]. Previous studies have reported elevated Aβ deposition in POS with the increasing age of the donor [15]. This is another potential route through which Aβ could enter the phagosome autophagy–lysosomal pathway of RPE cells, suggesting that the chronic Aβ-burden may in fact be more pronounced than hitherto suspected. Taken together, our findings show dynamic Aβ effects recapitulating AMD-like features in living eyes as well as novel disease-causing mechanisms at the single-cell level (Figure 9a–d), suggesting previously unreported roles for this molecule in retinal pathology.

## 5. Conclusions

AMD is a multifactorial disease with an incompletely defined etiology. Currently, this common cause of blindness, which affects millions of individuals globally, has no effective treatment. The accumulation of the Alzheimer’s disease-associated Aβ proteins reported in donor aged and AMD eyes has prompted studies to elucidate its mode of action in the retina. Our findings show hitherto unreported AMD-like features in living mouse eyes as well as direct Aβ effects in endothelial cells. Studies in the RPE revealed evidence of lysosomal dysfunction alongside conditions favoring the intracellular accumulation of Aβ. These insights suggest impairment of cellular proteostasis including clearance mechanisms in the RPE, which is typically associated with the intracellular accumulation of pathogenic macromolecules. Our discoveries alongside findings from other groups indicate that targeting Aβ synthesis and clearance mechanisms in the retina may be a promising pathway towards developing a new treatment for AMD. Hence, re-purposing of anti-Aβ compounds developed for Alzheimer’s disease could be a useful approach.

## Figures and Tables

**Table 1:** Primary and secondary antibodies used for confocal immunofluorescence microscopy studies. Antibodies were diluted in blocking buffer to ratios indicated in the table. Ig: immunoglobulin; mAb: monoclonal antibody; pAb: polyclonal antibody.

Product Name	Company	Catalogue Number	Clone/Isotype	Species	Dilution	RRID
ZO-1 Antibody	Invitrogen	40–2200	IgG, pAb	Rabbit	1:100	AB_2533456
Anti-human Amyloid β (N) (82E1)	IBL	10323	82E1, IgG1, mAb	Mouse	1:100	AB_10707424
Anti-β3-Tubulin	Abcam	ab18207	IgG, pAb	Rabbit	1:200	AB_444319
Anti-LAMP1	Abcam	ab24170	IgG, pAb	Rabbit	1:1000	AB_775978
Anti-Rhodopsin [RET-P1]	Abcam	ab3267	IgG1,mAb	Mouse	1:100	AB_303655
Anti-PSD-95	Cell Signaling Technologies	2507s	pAb	Rabbit	1:100	AB_561221
Anti-LRP1 [EPR3724]	Abcam	ab92544	IgG, mAb	Rabbit	1:500	AB_2234877
Anti-Rabbit Alexa Fluor^®^ 594	Life Technologies	A11072	IgG, pAb	Goat	1:200	AB_142057
Anti-Mouse Alexa Fluor^®^ 594	Life Technologies	A11020	IgG, pAb	Goat	1:200	AB_141974

**Table 2:** Quantitative Real-Time PCR primers. Sequences of forward (F) and reverse (R) primers used to amplify cDNA fragments in qPCR analysis. The corresponding gene is shown along with primer GC content, and expected amplicon length in base pairs. Primers were designed using PrimerBlast (NCBI) against the listed accession number for each gene and to span exon–exon junctions. CTSB: Homo sapiens cathepsin B; ACTB: Homo sapiens actin beta; GAPDH: Homo sapiens glyceraldehyde-3-phosphate dehydrogenase; CYC1: Homo sapiens cytochrome c1; EIF4A2: Homo sapiens eukaryotic translation initiation factor 4A2.

Gene	Accession	Sequence (5′–3′)	Tm (°C)	GC Content (%)	Amplicon Length (bp)
CTSB	NM_001908.4	F: GGGCCGGGAGGGTACTTA	60.0	66.7	145
		R: GATCCTAGATCCACCCAGCG	59.4	60.0	
ACTB	NM_001101.3	F: ACAGAGCCTCGCCTTTGCC	62.9	63.2	70
		R: GATATCATCATCCATGGTGAGCTGG	61.2	48.0	
GAPDH	NM_001289745.1	F: GAAGACGGGCGGAGAGAAAC	60.7	60.0	151
		R: CGACCAAATCCGTTGACTCC	58.9	55.0	
CYC1	NM_001916.4	F: TACGGACACCTCAGGCAGT	60.2	57.9	183
		R: CACGGTGAGACCACGGATAG	59.9	60.0	
EIF4A2	NM_001967.3	F: GGTCAGGGTCAAGTCGTGTT	59.9	55.0	136
		R: CCCCCTCTGCCAATTCTGTG	60.7	60.0	

**Table 3:** Effects of subretinal Aβ on global retinal function assessed by scotopic full-field electroretinography. Retinal function was assessed 1 and 2 weeks post-injection with either Aβ_1-42_ (*n* = 7) or vehicle (*n* = 6). Data shown as an average percentage change from baseline +/- the standard error of the mean (SEM) to account for intra-animal variability. Statistical significance was assessed between groups using the Mann–Whitney U test which showed no change in A-wave (*p* = 0.945, *p* = 0.988), B-wave (*p* = 0.534, *p* = 0.683), T_(A)_ (*p* = 0.445, *p* = 0.098) and T_(B)_ (*p* = 0.731, *p* = 0.564) recordings after 1 and 2 weeks, respectively. These findings suggest that subretinal Aβ accumulation does not significantly impact retinal function when measured across the whole retina.

ERG Component	Week 1		Week 2	
	Vehicle	Aβ	Vehicle	Aβ
**A Wave**	39.3 ± 40.2	25.7 ± 24.6	100.2 ± 71.3	98.8 ± 63.1
**B Wave**	−7.1 ± 19.6	4.7 ± 12.0	34.8 ± 32.1	53.5 ± 30.7
**T_(A)_**	−10.1 ± 9.4	7.2 ± 12.2	−17.4 ± 7.0	−0.8 ± 6.0
**T_(B)_**	−41.3 ± 19.3	−49.3 ± 14.3	−37.6 ± 19.9	−52.3 ± 15.2

**Table 4:** Effects of subretinal Aβ on the global thickness/structure of individual retinal layers as well as total retinal thickness assessed by optical coherence tomography. Measurements were carried out in mice subretinally injected with either Aβ_1-42_ (*n* = 7) or vehicle (*n* = 6) after 1 or 2 weeks. Data shown as an average percentage change from baseline +/- the standard error of the mean (SEM) to account for intra-animal variability. Statistical analysis using the Mann–Whitney U test revealed no change in the RNFL (*p* = 0.1375, *p* = 0.1807), GCLIPL (*p* = 0.3660, *p* = 0.5338), INL (*p* = 0.4452, *p* = 0.1014), OPL (*p* = 0.2343, *p* = 0.3660), ONL (*p* = 0.6282, *p* = 0.9452), IS (*p* = 0.3660, *p* = 0.7308), OS (*p* = 0.4452, *p* = 0.5338), ETPRS (*p* = 0.9452, *p* = 0.8357), RPE (*p* = 0.3660, *p* = 0.7308) or total RT (*p* = 0.7308, 0.9452) at week one and two, respectively. RNFL: Retinal Nerve Fiber Layer; GCIPL: Ganglion Cell Inner Plexiform Layer; INL: Inner Nuclear Layer; OPL: Outer Plexiform Layer; ONL: Outer Nuclear Layer IS: Inner Segments; OS: Outer Segments, ETPRS: End Tips of Photoreceptors; RPE: Retinal Pigment Epithelium; RT: total Retinal Thickness (the sum of all the layers measured).

Retinal Layer	Change in Layer Thickness (%)			
	Week 1		Week 2	
	Vehicle	Aβ	Vehicle	Aβ
**RNFL**	−9.2 ± 5.9	−22.9 ± 5.3	−21.9 ± 4.4	−31.8 ± 6.8
**GCL/IPL**	−7.0 ± 3	−4.4 ± 2.6	−10.7 ± 3.2	−7.2 ± 2.5
**INL**	7.1 ± 4.5	14.6 ± 4.8	2.5 ± 4.1	−4.7 ± 2.9
**OPL**	5.4 ± 9.4	−12.9 ± 10.4	−18.9 ± 10.9	13.0 ± 31.6
**ONL**	−2.1 ± 6.0	−0.06 ± 7.9	0.007 ± 8.0	−2.0 ± 6.2
**IS**	23.5 ± 14.7	3.65 ± 9.0	−4.0 ± 10.4	8.6 ± 10.6
**OS**	−2.8 ± 8.3	−9.6 ± 6.3	1.3 ± 9.5	−10.7 ± 8.0
**ETPRS**	−4.1 ± 13.1	−7.1 ± 7.6	−4.5 ± 13.8	−7.3 ± 7.7
**RPE**	10.3 ± 21.5	−14.2 ± 18.8	−5.5 ± 14.2	−22.4 ± 8.4
**RT**	−2.2 ± 2.4	−3.6 ± 2.1	−2.9 ± 1.6	−3.1 ± 2.1

## Data Availability

All data are included in this manuscript and in Supplementary Materials. Reasonable requests for raw data will be considered by the authors before being made available to third parties.

## Supplementary Materials

The following are available online at https://www.mdpi.com/2073-4409/10/2/413/s1, Supplementary information includes figures (Figures S1–S12) and movies of OCT images at baseline, week 1 and week 2 post-injection.
