# Supplementary material for: Oligomeric Aβ1-42 Induces an AMD-Like Phenotype and Accumulates in Lysosomes to Impair RPE Function
Source: Cells. 2021 Feb 17;10(2):413. doi: 10.3390/cells10020413 (PMC7922851; doi:10.3390/cells10020413)
Supplement: Supplementary file 1 [file cells-10-00413-s001.zip › Supplementary Material/Supplementary Information.pdf]

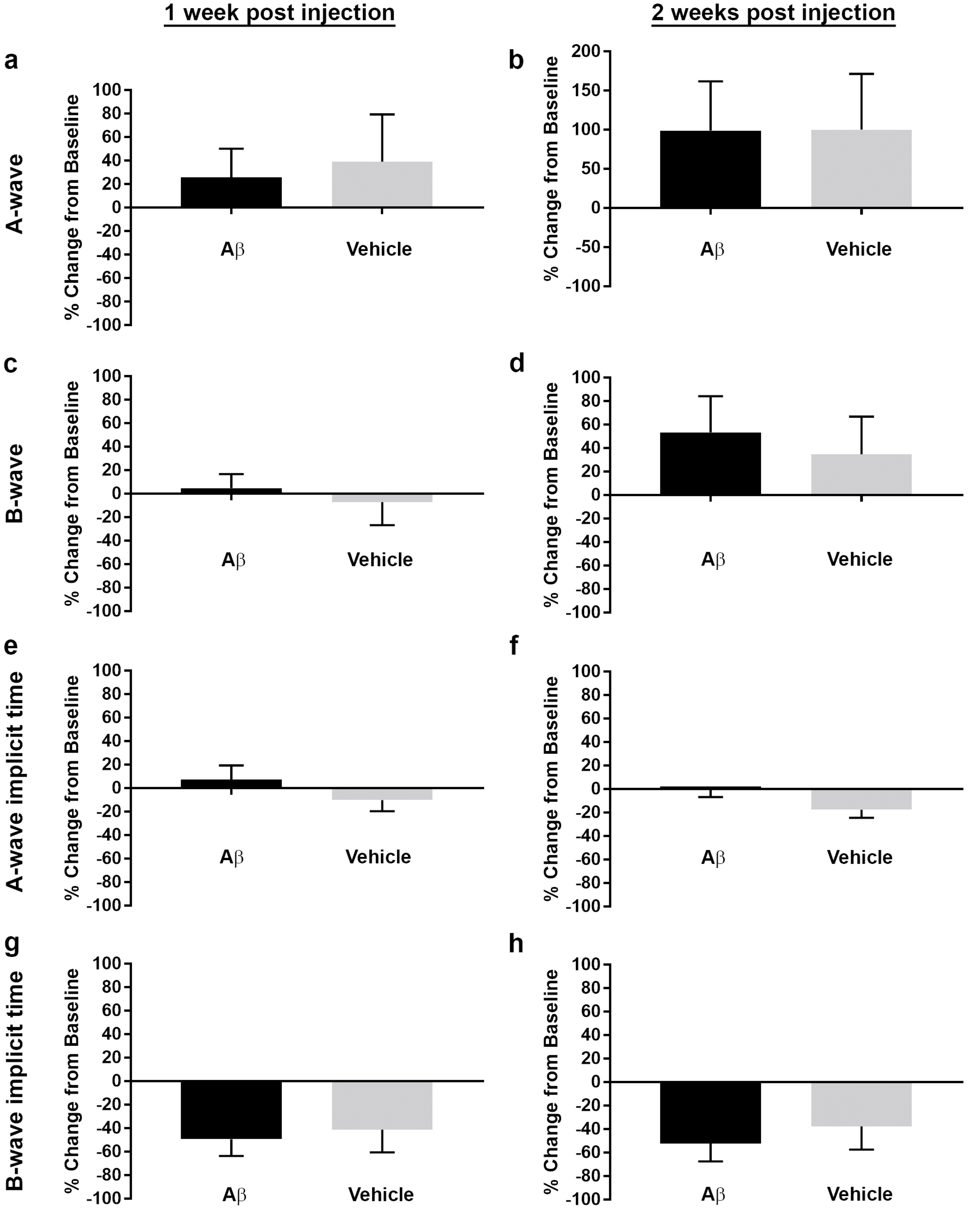

Supplementary Figure S1. Assesment of retinal function in mice injected with Aβ or vehicle after 1 and 2 weeks. Scotopic ERG recordings were carried out in mice injected with human oligomeric $A\beta_{1-42}$ (n=7) or vehicle (n=6) after 1 and 2 weeks. [a-b] Retinal A-wave and [c-d] B-wave functions as well as [e-f] A-wave implicit and [g-h] B-wave implict times. Data shown as a percentage change from baseline ERG values. A-wave (1 week: p=0.95, 2 weeks: p=0.96), B-wave (1 week: p=0.53, 2 weeks: p=0.68), $T_{(A)}$ (1 week: p=0.45, 2 weeks: p=0.1) and $T_{(B)}$ (1 week: p=0.73, 2 weeks: p=0.56) Mann-Whitney U test. No differences in global retinal function was observed between Aβ vs. control (vehicle injected) animals.

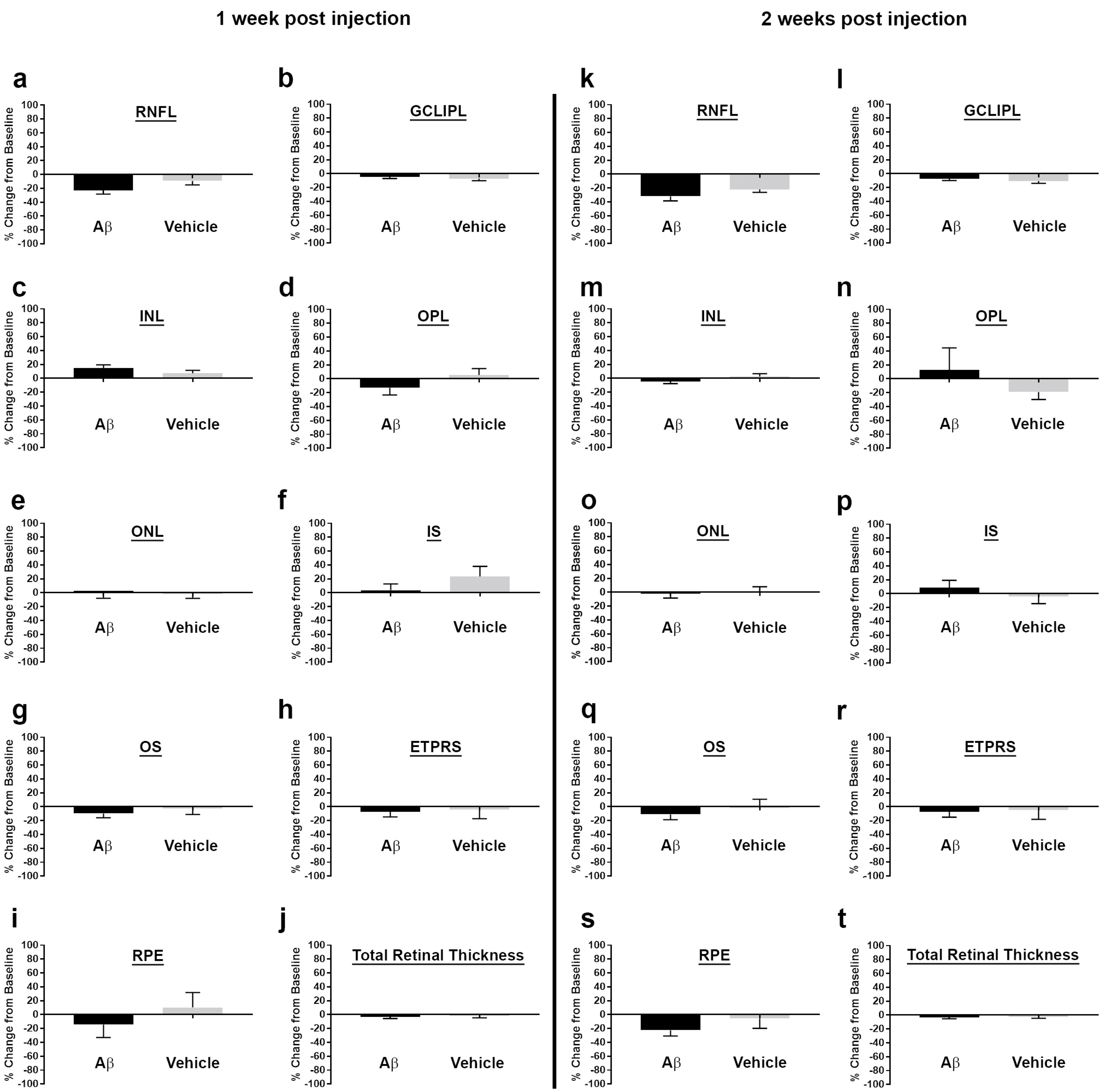

Supplementary Figure S2: Assesment of retinal structure in mice injected with Aβ or vehicle after 1 and 2 weeks. The structure of the retina and associated tissues in living mouse eyes were longitudinally assessed by optical coherence tomography (OCT). Eyes were injected with either human oligomeric $A\beta_{1-42}$ (n=7) or vehicle (n=6). Data shown as a percentage change from baseline OCT recordings for each animal. RNFL (week 1: p=0.14, week 2: p=0.18), GCLIPL (week 1: p=0.37, week 2: p=0.53), INL (week 1: p=0.45, week 2: p= 0.10), OPL (week 1: p=0.23, week 2: p=0.37), ONL (week 1: p=0.63, week 2: p=0.95), IS (week 1: p=0.37, week 2: p=0.73), OS (week 1, p=0.45, week 2: p=0.53), ETPRS (week 1: p=0.95, week 2: p=0.84), RPE (week 1: p=0.37, week 2: p=0.73), Total Retinal Thickness (week 1: p=0.73, week 2: p=0.95). Mann-Whitney U test. No gross diferences were observed in any ocular tissues between Aβ vs. control (vehicle injected) eyes. Retinal Nerve Fibre Layer (RNFL), Ganglion Cell Layer/Inner Plexiform Layer (GCLIPL), Inner Nuclear Layer (INL), Outer Plexiform Layer (OPL), Outer Nuclear Layer (ONL), Inner Segments (IS), Outer Segments (OS), End Tips of Photoreceptors (ETPRS), Retinal Pigment Epithelium (RPE).

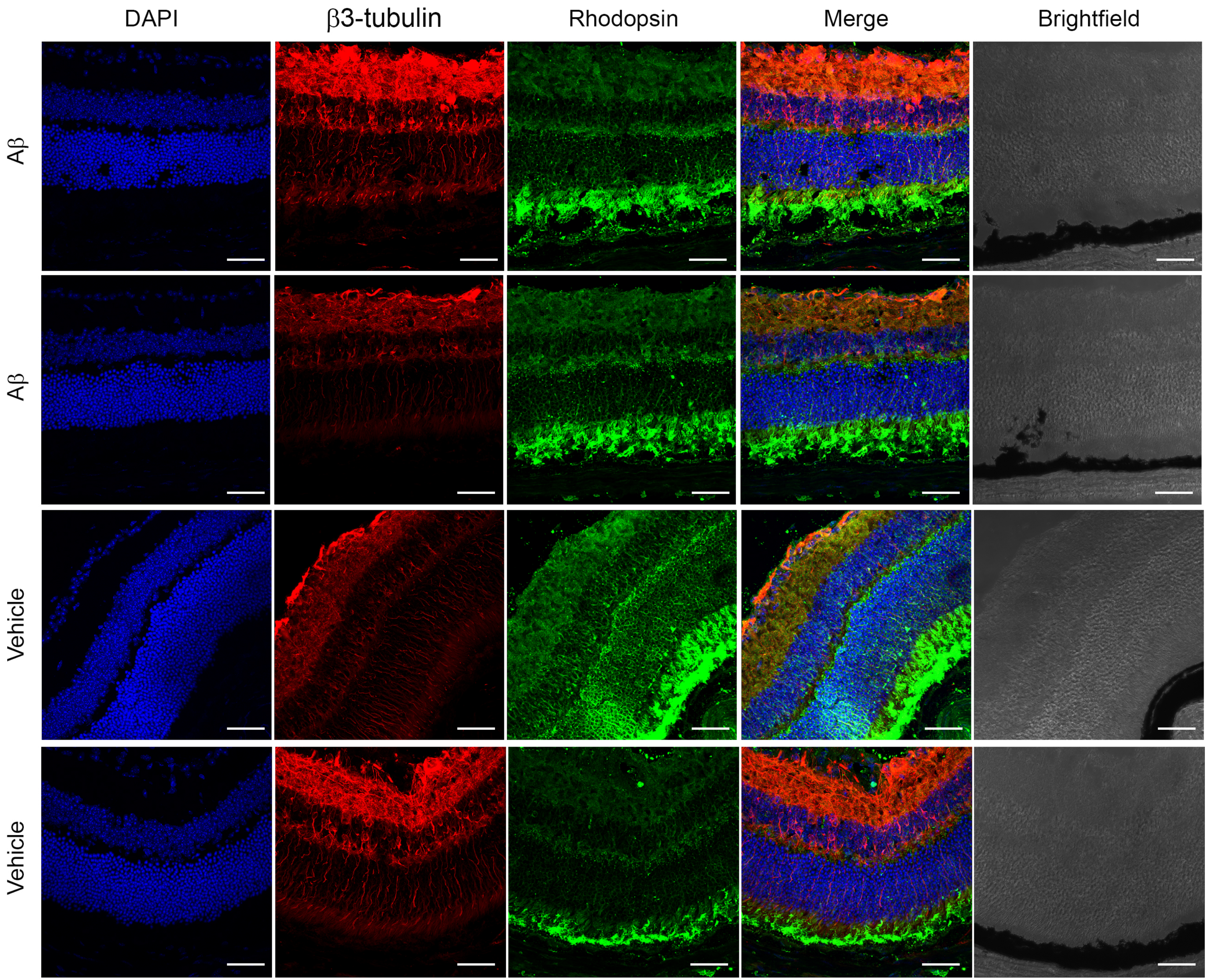

Supplementary Figure S3: AMD-like histopathology in the outer retina of mouse eyes exposed to human oligomeric A$\beta_{1\text{-}42}$ at one week post injection. Representitive images from seperate animals showing eyes injected with human oligomeric A$\beta_{1\text{-}42}$ or vehicle which were immunostained with DAPI (blue), β3-tubulin/TUJ-1 (red) and Rhodopsin (green), and anlyzed by confocal microscopy to determine potential changes to retinal architecture. Note, the extent of outer segment and RPE disruption in eyes exposed to Aβ compared to controls. Scale bars correspond to 40μm.

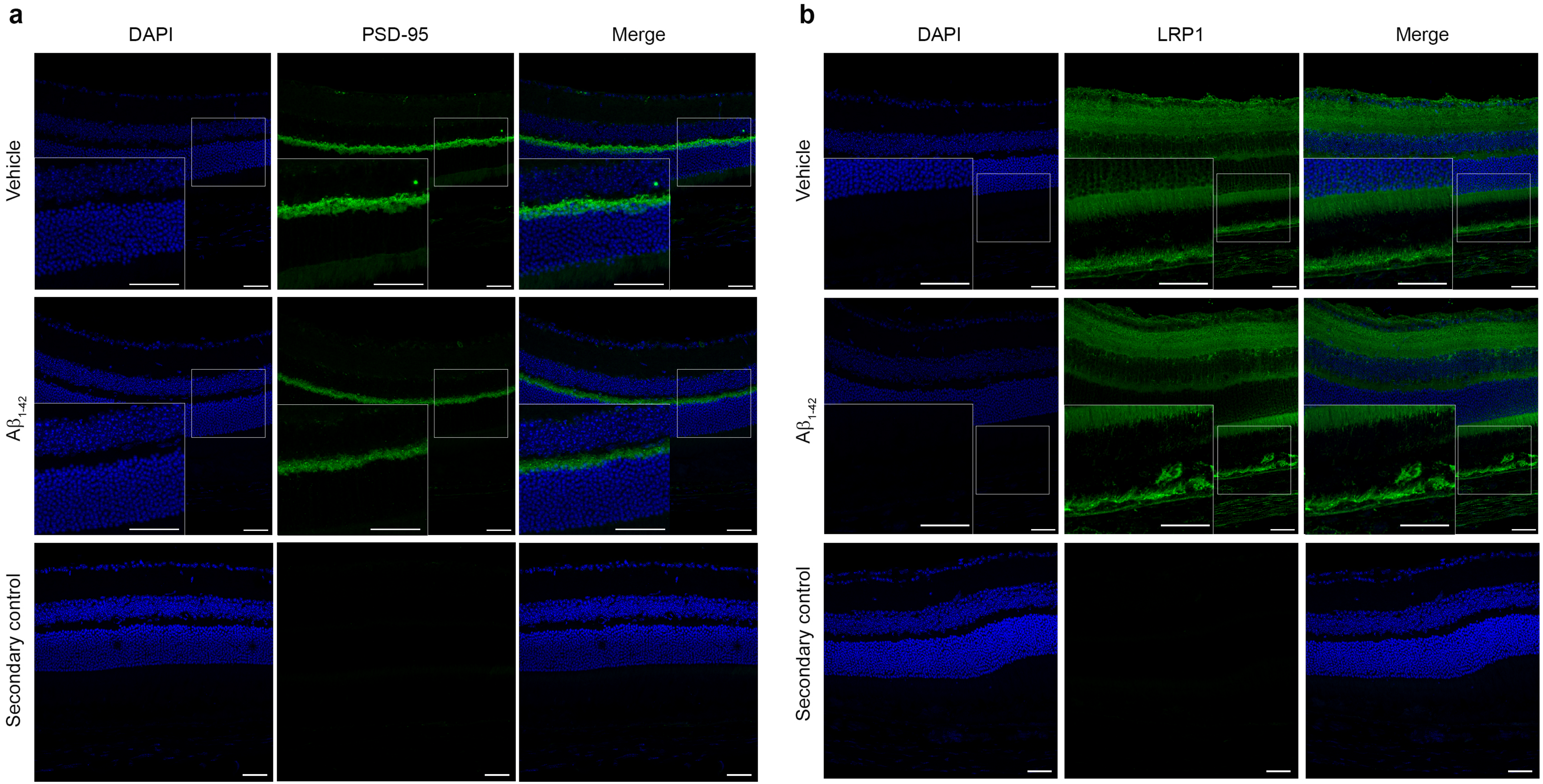

Supplementary Figure S4: Effects of subretinally injected Aβ on LRP1 and PSD-95 Expression. Mouse eyes injected with human oligomeric $A\beta_{1-42}$ (n=3) or vehicle (n=3) were analyzed to determine potential changes to expression of the Aβ clearance receptor LRP1 and the postsynaptic density marker PSD-95. Two weeks after subretinal injections, animals were culled and ocular cross-sections assessed by confocal immunofluorescence microscopy. (a) Representative images showing PSD-95 expression in Aβ vs. vehicle injected eyes (b) Representative images showing LRP1 expression in Aβ vs. vehicle injected eyes. Magnified inserts (lower left corner) show the extent to which Aβ affects PSD-95 and LRP1 staining. Scale bars correspond to 40μm.

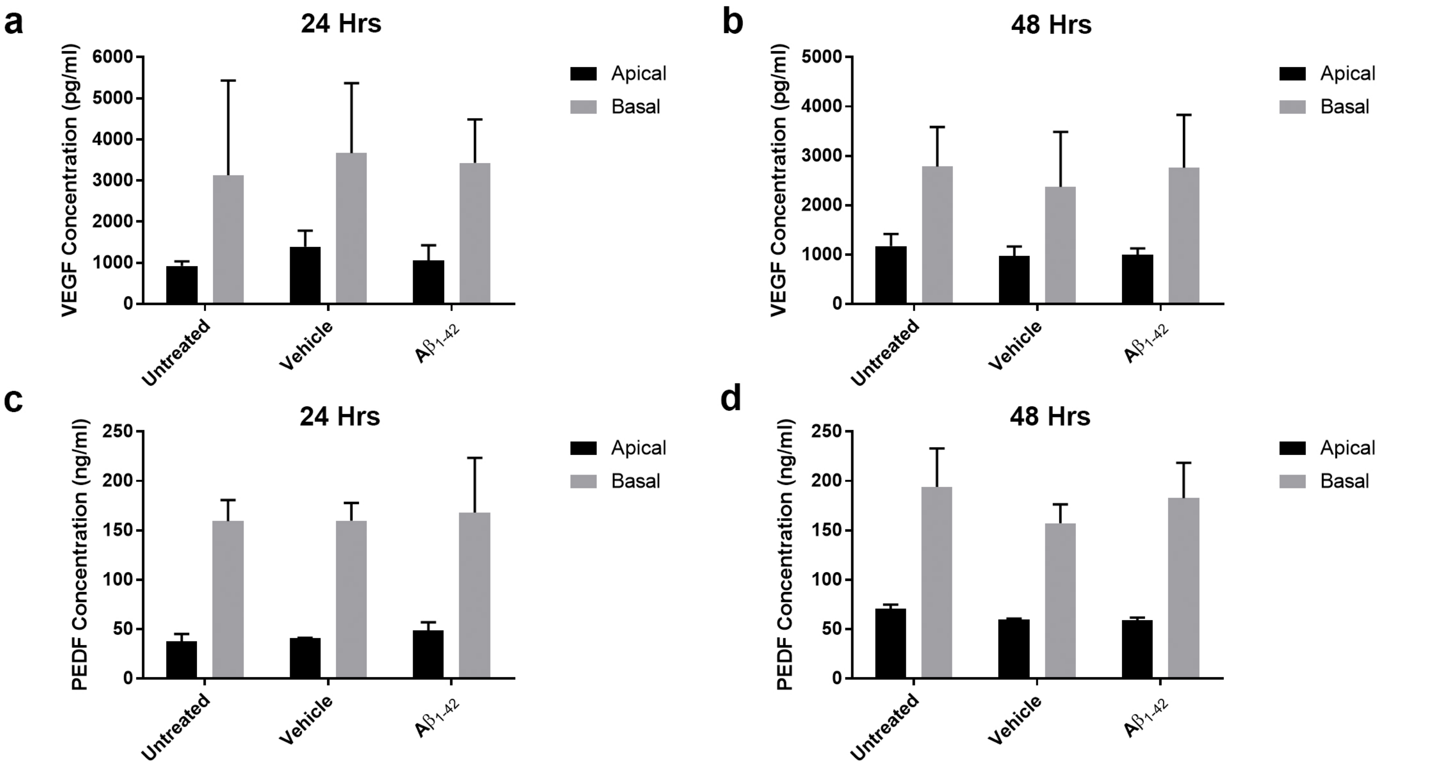

Time (Hours)	Treatment	PEDF Concentration (ng/ml)		VEGF Concentration (pg/ml)	
		Apical	Basal	Apical	Basal
24	Untreated	37.6 ± 7.5	159.2 ± 21.5	929 ± 78.1	1173 ± 174.6
	Vehicle	40.9 ± 0.5	159.9 ± 18.0	1393 ± 275.7	970.4 ± 137.8
	$A\beta_{1-42}$	48.6 ± 8.4	168.0 ± 55.4	1053 ± 266.5	997.9 ± 91.9
48	Untreated	70.4 ± 4.5	194.1 ± 38.9	3132.0 ± 1627	2787 ± 326.3
	Vehicle	60.1 ± 0.5	157.1 ± 19.2	3669 ± 1199.0	2374 ± 453.6
	$A\beta_{1-42}$	59.0 ± 2.8	182.7 ± 35.9	3435 ± 744.4	2759.0 ± 437.7

Supplementary Figure S5: Effect of Aβ on VEGF and PEDF levels secreted by cultured RPE cells. Cultures were treated with 1μM of human oligomeric $A\beta_{1-42}$ for 24 or 48 hours after which the quantity of VEGF and PEDF levels in apical and basal transwell compartments were quantified by ELISA (n=6, Kruskal-Wallis with Dunn's multiple comparisons tests). Data presented as S.E.M. No differences were observed between apically or basally secreted VEGF or PEDF levels in Aβ treated or untreated cultures.

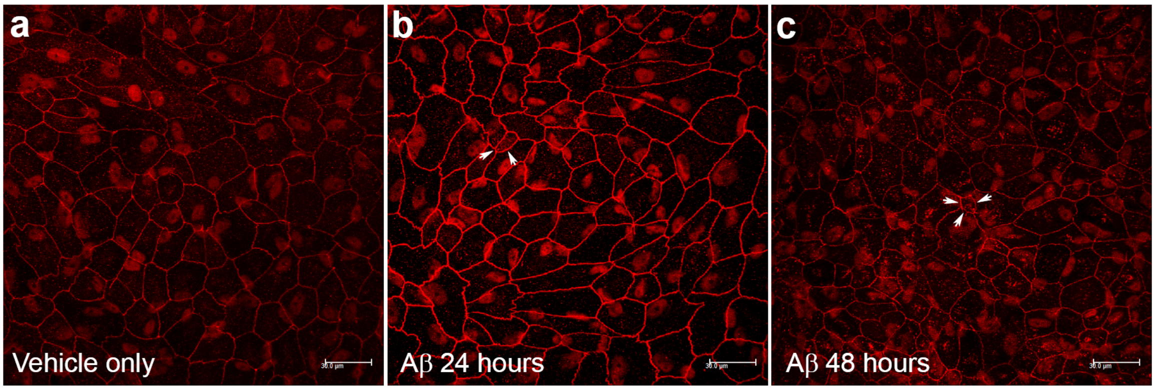

Supplementary Figure S6: Effects of Aβ on RPE cell morphology. Cells were treated with 750nM of human oligomeric $A\beta_{1-42}$ for 24 or 48 hours. Representitive confocal z-stacks labelled with ZO-1 revealed the presence of condensed cell foci (arrows) following Aβ exposure. Scale bars correspond to 30μm.

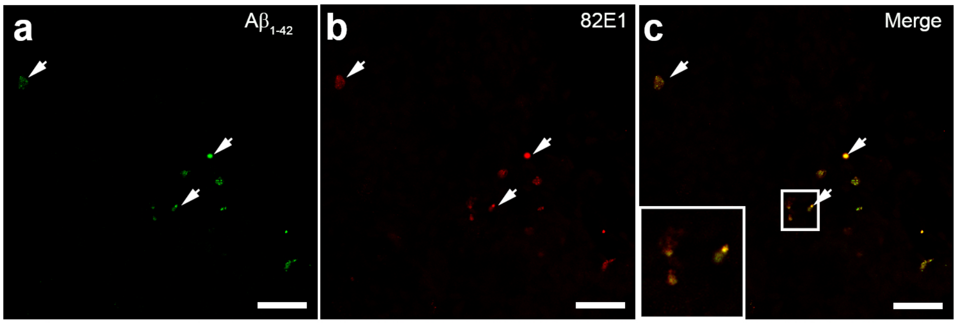

Supplementary Figure S7: Independent confirmation of fluorescently tagged Aβ molecules using an Aβ specific antibody. Confocal immunofluorescence studies show co-localization of [a] Alexa Fluor 488-tagged human $A\beta_{1-42}$ (green) with [b] anti-Aβ 82E1 antibody (red) as [c] yellow pucta (arrows). Representitive images from Aβ treated cultures after 48 hours. Endogenous Aβ in RPE cells are also detected by 82E1 and appear as only red. Scale bar corresponds to 40μm.

a

	**Mean** (μm)	**SD**
Untreated	0.68	+/- 0.05
Vehicle	0.64	+/- 0.06
$A\beta^-$	0.67	+/- 0.04
$A\beta^+$	0.81	+/- 0.07

b

	**Untreated**		**Vehicle**		**$A\beta^-$**		**$A\beta^+$**	
	**difference**	**(p value)**	**difference**	**(p value)**	**difference**	**(p value)**	**difference**	**(p value)**
Untreated	N/A		-0.0069		0.0138	0.97	-0.1312	0.003
Vehicle	0.007	0.996	N/A		0.021	0.92	-0.124	0.005
$A\beta^-$	-0.014	0.973	-0.02	0.92	N/A		-0.145	0.001
$A\beta^+$	0.131	0.003	0.12	0.005	0.145	0.001	N/A	

Supplementary Figure S8: Effect of Aβ on RPE lysosomal size. Values showing the diameter of LysoSensor-positive vesicles quantified in RPE cells that were untreated, treated with vehicle or human $A\beta_{1-42}$, 24 hours after Aβ exposure. (a-b) Results show a significant increase in vesicle dimeter in compartments containing Aβ compared to those in the same cell without Aβ cargo or in vehicle treated or untreated RPE cells.

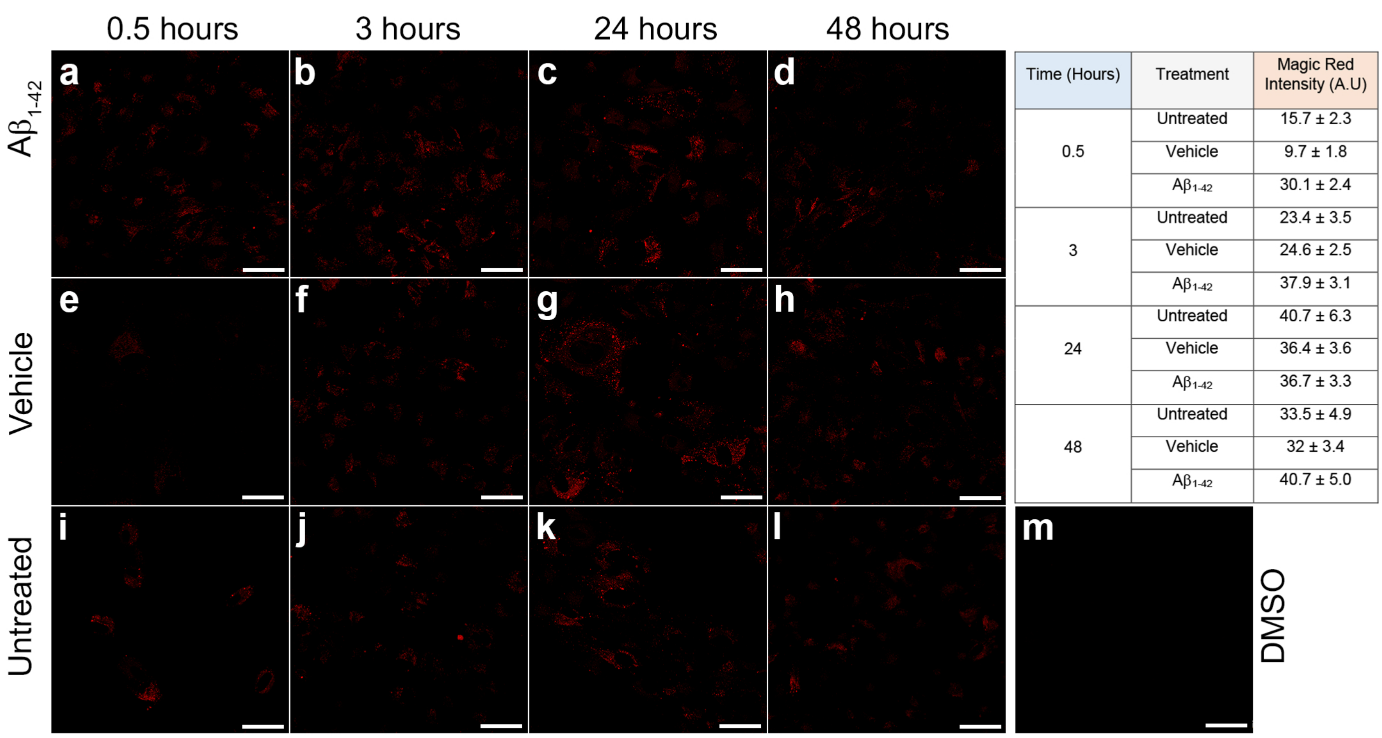

Time (Hours)	Treatment	Magic Red Intensity (A.U)
0.5	Untreated	15.7 ± 2.3
	Vehicle	9.7 ± 1.8
	$A\beta_{1-42}$	30.1 ± 2.4
3	Untreated	23.4 ± 3.5
	Vehicle	24.6 ± 2.5
	$A\beta_{1-42}$	37.9 ± 3.1
24	Untreated	40.7 ± 6.3
	Vehicle	36.4 ± 3.6
	$A\beta_{1-42}$	36.7 ± 3.3
48	Untreated	33.5 ± 4.9
	Vehicle	32 ± 3.4
	$A\beta_{1-42}$	40.7 ± 5.0

Supplementary Figure S9: Magic Red intensity in response to Aβ cargo. Representitive confocal images taken across single z-planes showing cathepsin B activity (indicated by the fluorescence intensity of Magic Red) in response to different treatments after 0.5, 3, 24 and 48 hours. n=40 (10 images across 4 independent experiments). Scale bars correspond to 40μm. Magic Red intensity presented as mean pixel intensity +/- SEM. Statistical significance assessed by Kruskal-Wallis with Dunn's multiple comparisons where $p<0.05$ is denoted by * and $p<0.0001$ is indicated by ****.

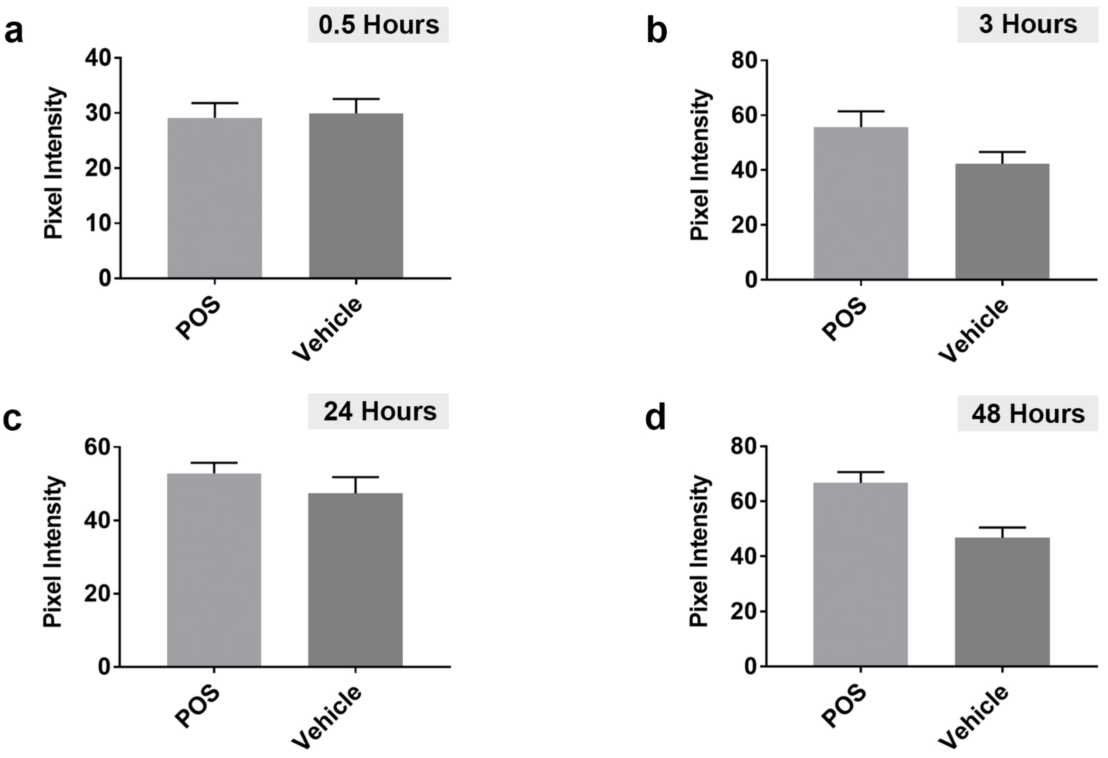

Supplementary Figure S10: Magic Red intensity in response to POS cargo.
In a sub-set of experiments, we studied the dynamics of lysosomal cathepsin B responses to internalised POS cargos in late endocytic compartments of RPE cells. Although we noted elevated enzymatic responses to cargos from the 3 hour time point onwards, these were not significantly different from baseline activity. Measurements in n=10 images across one experiment. Mean values with SEM.

a

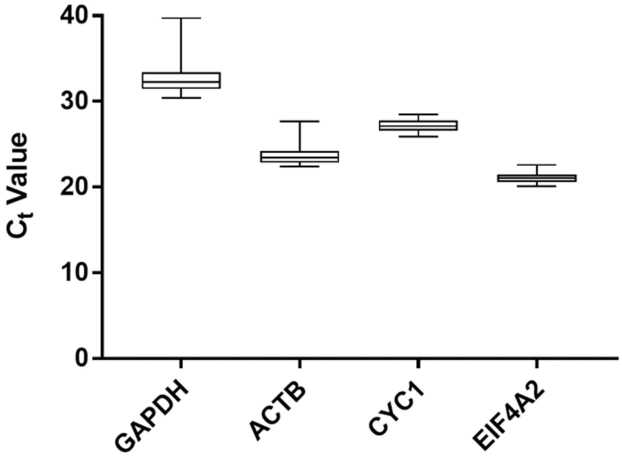

b

Time (Hours)	ΔCt					Fold Change ($2^{-\Delta\Delta CT}$)		
	Untreated	Vehicle	Aβ1-42			Vehicle	Aβ1-42	
				p value	F value			p value
0.5	15.2 ± 0.4	14.6 ± 0.8	15.9 ± 0.4	0.37	$F_{2,6}$=0.19	1.9 ± 0.9	0.7 ± 0.04	0.23
3	15.1 ± 0.5	15.2 ± 0.2	15.5 ± 0.6	0.86	$F_{2,6}$=0.15	1.2 ± 0.6	1.0 ± 0.4	0.76
24	15.2 ± 0.2	16.0 ± 0.5	15.4 ± 0.5	0.40	$F_{2,6}$=1.07	0.6 ± 0.2	1.1 ± 0.6	0.46
48	15.7 ± 0.1	15.7 ± 0.9	16.2 ± 0.5	0.79	$F_{2,6}$=0.25	1.3 ± 0.6	0.8 ± 0.2	0.47

Supplementary Figure S11: Cycle threshold (C_t) values for candidate reference genes. We sought to determine the expression level of lysosomal cathepsin B mRNA in response to different treatments. However, a realiable reference gene with stable mRNA levels in cultured RPE treated with Aβ had to be first indentified. [a] Graph shows C_t values for candidate reference genes glyceraldehyde-3-phosphate dehydrogenase (GAPDH), β-actin (ACTB), cytochrome c1 (CYC1) and the eukaryotic translation initiation factor 4A (EIF4A2), n=36 from 3 biological replicates. Graphs are represented as medians (line), 25th percentile to the 75th percentile (boxes) and ranges (whiskers). EIF4A2 showed the least variability and was selected as the reference gene against which cathepsin mRNA levels were subsequently compared. [b] ΔC_t scores reporting cathepsin B mRNA levels +/- SEM in relation to EIF4A2 (one-way ANOVA with Tukey's multiple comparisons test) and fold change in expression +/- SEM (two-tailed unpaired Student's t-test). Reported p values for ΔC_t are for one-way ANOVA.

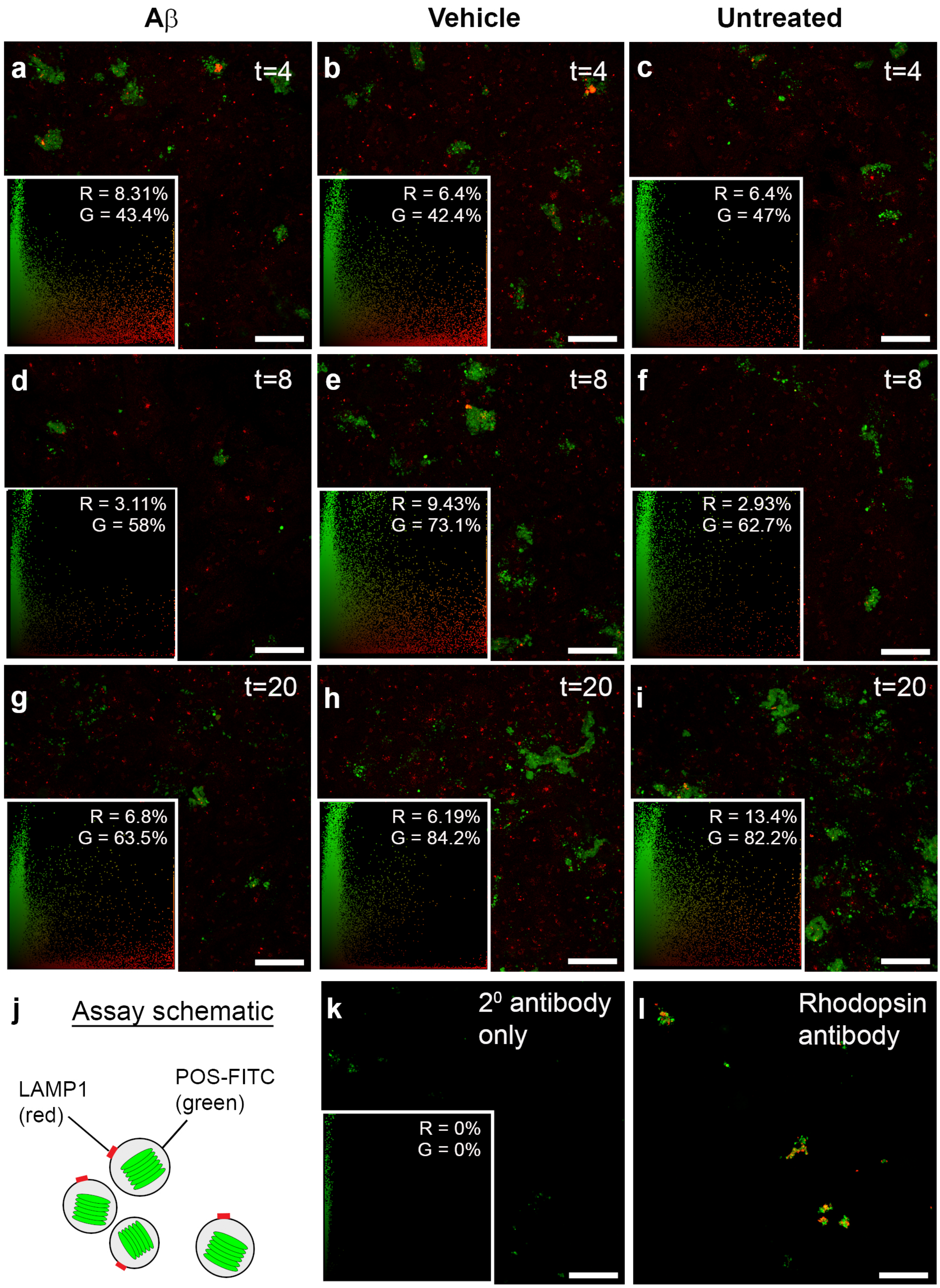

Supplementary Figure S12: Assesment of POS trafficked into RPE lysosomes. Representitive images showing POS-FITC (green) co-localizing with LAMP1 positive lysosomes (red) following a feeding assay. Inset scatter plots denote % of co-labelling. Note, reduced extent of green (G) co-localising with red (R) at 8 (d-f) and 20 hour time points (g-i) with Aβ. Time (t) in hours after feeding assay. Images from duplicate wells in 2 biological replicates. POS labelling was validated using a rhodopsin antibody; co-localization in yellow (k-l). Scale bars correspond to 40μm.
